# Mesenchymal stem cell-derived exosomes protect against liver fibrosis via delivering miR-148a to target KLF6/STAT3 pathway in macrophages

**DOI:** 10.1186/s13287-022-03010-y

**Published:** 2022-07-20

**Authors:** Siyuan Tian, Xia Zhou, Miao Zhang, Lina Cui, Bo Li, Yansheng Liu, Rui Su, Keshuai Sun, Yinan Hu, Fangfang Yang, Guoyun Xuan, Shuoyi Ma, Xiaohong Zheng, Xinmin Zhou, Changcun Guo, Yulong Shang, Jingbo Wang, Ying Han

**Affiliations:** 1 State Key Laboratory of Cancer Biology, Xijing Hospital of Digestive Diseases, The Fourth Military Medical University, Xi’an, 710032 Shaanxi China; 2 Department of Gastroenterology, The Air Force Hospital From Eastern Theater of PLA, Nanjing, 210002 Jiangsu China

**Keywords:** Liver fibrosis, Mesenchymal stem cells, Exosome, miR-148a, Macrophage polarization

## Abstract

**Background:**

Despite emerging evidence on the therapeutic potential of mesenchymal stem cells (MSCs) for liver fibrosis, the underlying mechanisms remain unclear. At present, MSC-derived exosomes (MSC-EXOs) are widely accepted as crucial messengers for intercellular communication. This study aimed to explore the therapeutic effects of MSC-EXOs on liver fibrosis and identify the mechanisms underlying the action of MSC-EXOs.

**Methods:**

Carbon tetrachloride was used to induce a liver fibrosis model, which was intravenously administered with MSCs or MSC-EXOs to assess treatment efficacy. The resulting histopathology, fibrosis degree, inflammation and macrophage polarization were analyzed. RAW264.7 and BMDM cells were used to explore the regulatory effects of MSC-EXOs on macrophage polarization. Then, the critical miRNA mediating the therapeutic effects of MSC-EXOs was screened via RNA sequencing and validated experimentally. Furthermore, the target mRNA and downstream signaling pathways were elucidated by luciferase reporter assay, bioinformatics analysis and western blot.

**Results:**

MSCs alleviated liver fibrosis largely depended on their secreted exosomes, which were visualized to circulate into liver after transplantation. In addition, MSC-EXOs were found to modulate macrophage phenotype to regulate inflammatory microenvironment in liver and repair the injury. Mechanically, RNA-sequencing illustrates that miR-148a, enriched in the MSC-EXOs, targets Kruppel-like factor 6 (KLF6) to suppress pro-inflammatory macrophages and promote anti-inflammatory macrophages by inhibiting the STAT3 pathway. Administration of miR-148a-enriched MSC-EXOs or miR-148a agomir shows potent ameliorative effects on liver fibrosis.

**Conclusions:**

These findings suggest that MSC-EXOs protect against liver fibrosis via delivering miR-148a that regulates intrahepatic macrophage functions through KLF6/STAT3 signaling and provide a potential therapeutic target for liver fibrosis.

**Supplementary Information:**

The online version contains supplementary material available at 10.1186/s13287-022-03010-y.

## Background

Liver fibrosis is chronic liver damage characterized by excessive accumulation of extracellular matrix [1]. It is a global health problem that affects millions of people annually worldwide [2]. Without proper treatment, many patients will suffer from the compensation stage to the decompensation stage, which increases morbidity and mortality caused by portal hypertension, hepatic insufficiency, and other complications [3]. Various therapies have been explored to treat patients with end-stage liver diseases aiming at promoting liver regeneration and alleviating liver injury.

Recently, mesenchymal stem cells (MSCs) have attracted much attention for potential treatment due to their differentiation ability and immunomodulatory effect. Emerging research and clinical trials are ongoing for the application of MSCs as a regenerative solution for many diseases including spinal cord injury [4], organ fibrosis [5], inflammatory bowel disease [6] and graft-versus-host disease [7]. Now, paracrine actions of MSCs are thought to be an important therapeutic way. For example, application of MSC-conditioned medium (MSC-CM) can improve liver cirrhosis via Milk Fat Globule-EGF Factor 8 to downregulate expression of TGFβ type I receptor by binding to αβ integrin on hepatic stellate cells [8]. Our previous study also illustrated that tumor necrosis factor-α-stimulated gene 6 (TSG-6) derived from MSCs can improve liver fibrosis by modulating M2 macrophages and increasing matrix metalloproteinase 12 (MMP12) expression [9]. Moreover, several molecules derived from MSC-CM showed immunoregulatory role, such as indoleamine-2,3-dioxygenase (IDO), prostaglandin E2 (PGE2) and human leukocyte antigen (HLA)-G [10]. Although these studies provide inspiring evidence for the potential role of MSC-secreted molecules in tissue regeneration, the detailed mechanisms through which MSCs act in a paracrine way are not fully understood.

Over the past years, in addition to soluble factors, extracellular vesicles especially exosomes (EXOs) have been demonstrated to be essential paracrine components of MSCs [11]. EXOs are small membrane particles ranging from 40 to 150 nm in size, which are regarded as a crucial factor in communication between cells or organs [12]. EXOs contain numerous bioactive molecules including proteins, mRNAs and non-coding RNAs like microRNAs, which can be functionally delivered between cell types, even across species [13]. Exosomal miR-192-5p derived from hepatocyte was found to play a critical role in the activation of pro-inflammatory macrophages and disease progression through modulating Rictor/Akt/FoxO1 signaling [14]. Moreover, hypoxia-preconditioned MSCs prevented ischemic disease by promoting angiogenesis via miR-612 transfer [15]. It has also been shown that exosomes derived from MSCs possess comparably therapeutic effect and offer an alternative for cell-based therapy [16, 17].

In this study, we demonstrated that MSCs-derived exosomes (MSC-EXOs) alleviated carbon tetrachloride (CCL4)-induced liver fibrosis in mice. By employing high-throughput sequencing, miR-148a was identified to be a pivotal factor of MSC-EXOs, which was also proved to participate in liver regeneration in our previous research [18]. Here, miR-148a was verified to reshape macrophage polarization and alleviate liver fibrosis. Then, mechanistic studies showed that miR-148a acted by downregulating Kruppel-like factor 6 (KLF6) to inhibit the signal transducer and activator of transcription 3 (STAT3) pathway. Afterward, we administered miR-148a-enriched MSC-EXOs or miR-148a agomir to reveal its therapeutic effects on liver fibrosis, which indicated the translational potential for the management of liver diseases.

## Methods

### Patients and clinical samples

Serum samples were collected from 24 patients with liver cirrhosis and 12 healthy controls at the Xijing Hospital of Digestive Disease. The diagnosis of cirrhosis followed the American Association for the Study of Liver Disease (AASLD) guideline [19]. The aspartate aminotransferase (AST)- to platelet ratio index (APRI) score [20] and Fibrosis-4 (FIB-4) score [21] were calculated according to relevant formulas. Informed consent was obtained from all participants, and the study protocol was approved by the Ethics Committee of Xijing Hospital. The detail information of patients is listed in Additional file 1: Table S1.

### Experimental animal models

The male C57BL/6 J mice (6–8 weeks) were brought from and cared in animal center of The Fourth Military Medical University. The animal study protocol was approved by the Animal Welfare and Ethics Committee of the Fourth Military Medical University and performed according to the “Guidelines for the Care and Use of Laboratory Animals”. The liver fibrosis model was established via intraperitoneal injection of 0.2 ml/20 g 20% (v/v) of CCL4 for 8 weeks twice a week. After then, the fibrotic mice were randomly into groups (*n* = 4–6) which received treatments as mentioned below via tail vein injection. The mice were continually injected with CCL4 during the observation period. After 2 weeks, mice were killed, and samples were collected for further analyses.

To investigate the role of MSC-EXOs in the liver fibrosis, MSC were pre-treated with 10 μM GW4869 (Sigma Aldrich) for 24 h to inhibit exosomes release. Mice were then randomly assigned to different groups which received PBS, 1 × 10^6^ MSCs or pre-treated MSCs (PBS group, MSC group and MSC + GW4869 group). For exosome treatment, 150 μg of EXOs diluted in 150μL PBS was injected intravenously to mice via tail vein as a single dose. The control group was injected with an equal volume of PBS. In addition, we further expanded the treatment groups, that is, the fibrotic mice were treated with EXOs derived from MSCs transfected with miR-148a-3p mimics (EXO-148 m group) or inhibitors (EXO-148i group). For miRNA agomir injection, mice were injected with negative control (NC) agomir (NC-agomir) or miR-148a agomir (148a-agomir group) via tail vein injection at a dose of 5 nmol for 2 weeks, once per week.

### Cell culture

Human umbilical cord-derived MSCs (hUC-MSCs) were provided by the National Engineering Research Center (Tianjin AmCellGene Engineering Co., Ltd, China). The isolated MSCs were cultured with mesenchymal basal medium (Dakewe Biotech Co., Ltd. China) supplemented with a serum-free replacement at 37 °C in a 5% CO2 incubator. Cells in passage 4–7 were used for subsequent experiments. The positive (CD29, CD44, CD90 and CD105) and negative (CD34, CD45) cell surface markers of MSCs were identified by flow cytometer. Meanwhile, the multilineage differentiation potential of MSCs was tested in vitro for adipogenesis, osteogenesis as well as chondrogenesis (HUXUC-90021, HUXUC-90031, HUXUC-9004, Guangzhou, China).

RAW264.7 cells purchased from American Type Culture Collection (ATCC) were cultured in Dulbecco’s modified Eagle’s medium (DMEM, Gibco, NY) containing 10% fetal bovine serum (FBS, Gibco, NY) and 1% penicillin–streptomycin. Bone morrow-derived macrophages (BMDM) were generated from C57 BL/6 mice (6–8 week) as previously described [22]. In brief, bone marrow cells were harvested from the femur and tibia. Then, these cells were seeded at a density of 2 × 10^6^ cells/mL and cultured in DMEM supplemented with 10% fetal bovine serum and 1% penicillin–streptomycin plus 20 ng/mL macrophage colony-stimulating factor (M-CSF, PeproTech Inc. USA) for 7 days.

### Exosomes’ isolation and characterization

EXOs of hUC-MSCs were isolated and purified by differential centrifugation as previously described [23]. Briefly, when the cells have reached 70–80% confluence, the medium was replaced with the exosome-depleted medium (Umibio, Shanghai, China), and the cell supernatant was collected after additional 48 h. The cells and apoptotic bodies were removed after centrifugation at 300 g for 10 min and 2000 g for 20 min, respectively. The sample was then centrifuged at 10,000 g for 30 min to remove cell debris. Finally, the supernatant was centrifuged at 100,000 g at 4 °C for 70 min and additionally washed with PBS at 100,000 × g for 70 min to obtain exosome pellets. The EXOs were characterized by transmission electron microscopy (TEM), and their morphology was observed. Nanoparticle tracking analysis (NTA) was used to analyze the size distribution of EXOs and the concentration of nanoparticles. Flow cytometry was employed to detect exosome surface markers CD9 and CD81 (BioLegend, San Diego). Specific exosome markers (CD9, CD81 and TSG101) were used as a positive control, whereas GM130 was used as a negative control for western blot.

### Exosome labeling and tracking in vivo and ex vivo

To detect the uptake of MSC-EXOs by macrophages, EXOs were incubated with 1 μM PKH26 (Sigma-Aldrich, MA, USA) for 10 min at 37 °C followed by centrifuged at 100,000 g for 70 min to remove unbounded dye. The labeled EXOs were then co-cultured with RAW 264.7 or BMDM for 6 h. After treatment, cells were washed twice with PBS and fixed with 4% paraformaldehyde. Following DAPI staining, the cells were observed under a fluorescence microscope.

For in vivo exosome tracking, 1 μM DiR (Invitrogen, MA, USA) solution was used to label EXOs at 37 °C for 30 min. Free dyes were removed by ultracentrifugation as mentioned above. The supernatant was collected as the control. Mice that received labeled EXOs or supernatant via tail-vein injection were killed at 24 h. The distribution of fluorescence in the whole body and relative organs (heart, liver, spleen, lungs and kidneys) was detected using small animal imaging system (IVIS Kinetics, Caliper Life Science). Signal intensity was quantified and processed using the Living Image software (V5.0, Caliper Life Science).

### Cell treatment

For phenotype induction, the M1 (classically activated) and M2 (alternatively activated) phenotypes were established by stimulating RAW 264.7 cells or BMDMs with LPS (100 ng/mL)/IFN-γ (20 ng/mL) and IL-4 (20 ng/mL) for 24 h, respectively. To investigate the effects of hUC-MSC-derived EXOs on macrophage polarization, macrophages were treated with PBS or EXOs for 30 min prior to the M1 induction. Then, the cells were collected for western blot, real-time PCR or flow cytometry analysis.

### Cell transfection and luciferase report assay

The mimics and inhibitors of miR-148a-3p, NC mimics or inhibitor were purchased from Guangzhou RiboBio Co., Ltd. KLF6 siRNA and its NC siRNA were purchased from Sangon Biotech Co., Ltd. Cells were transfected using the riboFECT CP Transfection Kit (RiboBio) according to the manufacturer’s protocol. For the luciferase reporter assay, the 3′-UTR sequence of KLF6 (wild type) containing the possible sites binding with miR-148a-3p and the corresponding mutated 3′-UTR sequence were cloned and inserted into the pMIR-REPORT vector to generate luciferase reporter constructs. RAW264.7 cells were co-transfected with wild-type or mutated luciferase vector together with miR-148a-3p mimics and negative controls. The luciferase activity was measured using a luciferase reporter assay system (Promega, WI, USA).

### RNA-sequencing and bioinformatics analysis

The RNA-sequencing technology was provided by LC-Bio Co., Ltd. In brief, total RNA was extracted from MSC-EXOs using the TRIzol reagent. The purity and quantity of RNA were checked using a Bioanalyzer 2100 (Agilent, CA, USA). Small-RNA libraries were constructed using the TruSeq Small RNA Sample Prep Kits (Illumina, San Diego, CA USA) and sequenced on the Illumina HiSeq 2500 platform. Then, we downloaded the public data (GSE159814) from Gene Expression Omnibus (GEO) database and obtained the intersections with our sequencing data for further analysis. The potential target genes of miR-148a-3p were obtained through examination of the overlapped intersection from four databases (miRanda [24], miRDB [25], TargetScan [26] and CLIP [27]). In addition, to further investigate the signaling pathways that KLF6 was involved in, we performed pathway analysis using gene set enrichment analysis (GSEA) and gene set variation analysis (GSVA). Data were retrieved from GSE13476 in the GEO platform. A *P* value < 0.05 was considered statistically significant.

### Flow cytometric analysis of cell phenotypes

For quantification of macrophage infiltration, single-cell suspensions from liver tissues were treated with anti-mouse FcR blocking reagent and stained with mixed fluorescence-conjugated antibodies. The detail information of antibodies for flow cytometric is listed in Additional file 1: Table S2. Flow cytometric data were acquired on a FACSVerse flow cytometer (BD Bioscience, CA, USA) and analyzed with FlowJo software (TreeStar, Ashland, OR, US). Gating strategies are depicted in Additional file 1: Fig. S1.

### Biochemical analysis and histological staining

The serum of mice was obtained at each time point. The levels of alanine aminotransferase (ALT), aspartate aminotransferase (AST) and albumin (ALB) were analyzed on an automatic biochemistry analyzer in Xijing Hospital. Tissue samples were prepared as paraffin-embedded sections by YiKE Biotechnology, China. Then, sections were stained with hematoxylin and eosin (HE) for routine histological examination or with Sirius Red and Masson for fibrosis evaluation. Further quantification and analysis of collagen fiber were assessed using Image-Pro Plus software (v6.0, Media Cybernetics Inc.).

### Immunohistochemistry staining

For immunohistochemical analyses, the sections were dewaxed and rehydrated by xylol and alcohol, respectively. Antigen retrieval was conducted by sodium citrate in microwave condition. After then, 1% H_2_O_2_ was used to block endogenous peroxidase activity. The slides were further blocked with goat serum for 30 min. The antibodies of anti-iNOS (AB15323, 1:1000, Abcam, MA) and anti-CD206 (AB64693, 1:1000; Abcam, MA) were incubated with the slides at 4 °C overnight. On the next day, after washing with PBS, the secondary antibody was added and diaminobenzidine (DAB) was applied to visualize the image. Further quantification and analysis of positive area were assessed using Image-Pro Plus software. (v6.0, Media Cybernetics Inc.)

### Immunofluorescent staining

Liver tissue samples were fixed with 4% paraformaldehyde for 30 min and washed with PBS. After antigen retrieval with sodium citrate in microwave condition, the sections were blocked with goat serum for 30 min. Then, primary anti-iNOS antibody (AB15323, 1:200, Abcam), anti-CD206 antibody (AB64693, 1:200, Abcam), anti-F4/80 antibody (AB6640,1:100, Abcam), anti-αSMA antibody (A2547, 1:500, Sigma) and anti-Col1a1 (AB270993,1:100, Abcam) were added and incubated with tissue sections at 4 °C overnight. On the next day, the sections were washed three times with PBS and incubated with secondary antibody labeled with Alexa Fluor 488 or Alexa Fluor 647 (Invitrogen Inc.) at room temperature for 1 h. After DAPI staining of cell nucleus, sections were mounted using anti-fade solution. Later, images were acquired by a laser scanning confocal microscope. Further analysis of positive percentage cells was conducted by Image-Pro Plus software (v6.0, Media Cybernetics Inc, Bethesda, MD).

### RNA isolation and real-time PCR analysis

Total RNA was extracted by RNAeasy Plus kit (TaKaRa Biotechnology Co., Ltd., Dalian, China), and reverse transcription was performed using PrimeScript™ RT Master Mix (RR036A, Takara, Tokyo). Then, amplification was conducted by TB Green Premix Ex Taq II (DRR820A, Takara, Tokyo) on a CFX96 Touch™ real-time PCR System (Bio-Rad, CA). PCR primers are shown in Additional file 1: Table S3.

### Western blot analysis

Proteins of cells or tissue samples were obtained by RIPA lysis buffer (Beyotime biotechnology, China), which was added in proteinase inhibitors and phosphatase inhibitors (Roche, Basel, Switzerland). The Bradford method was applied to quantify the protein samples. After then, 30 μg of protein was loaded to SDS-PAGE before transferring to nitrocellulose membranes (Bio-Rad Biotechnology, America). The membranes were blocked by TBST buffer containing 2.5% skim milk for 30 min. Then, the membranes were incubated with primary antibody at 4 °C overnight. Next day, the peroxidase-conjugated secondary antibody was added to incubate the membrane, and blot images were acquired by an enhanced chemiluminescence kit.

### Statistical analysis

The data were in the expression of mean values ± standard deviation. One-way analysis of variance and *t*-test were performed to identify the significant differences. A *P* value < 0.05 was considered significant. Statistical analysis was plotted by GraphPad Prism 7.0 (GraphPad Software, CA, USA).

## Results

### Characterization of hUC-MSCs and EXOs

**Fig. 1:**
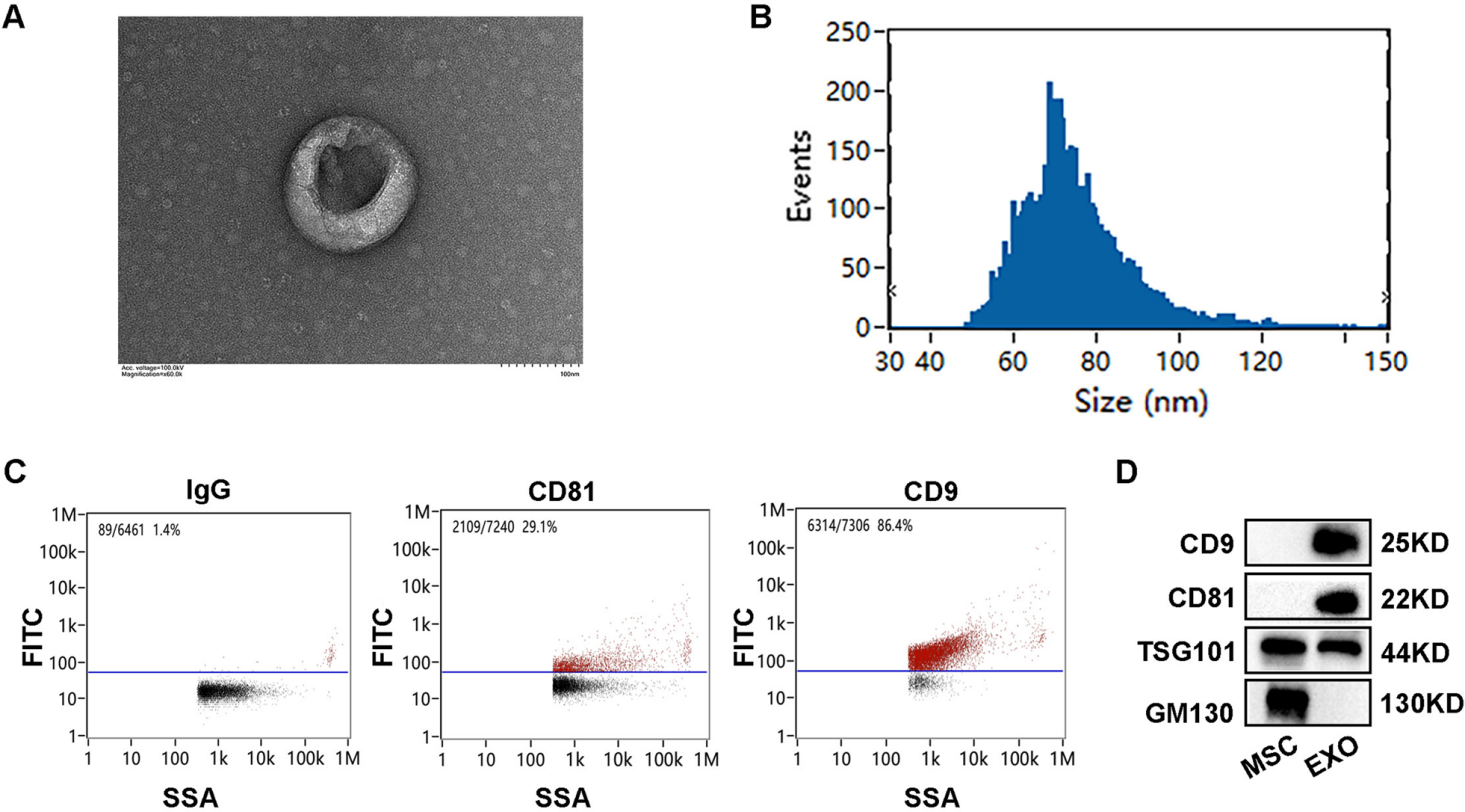
Identification of exosomes (EXOs) isolated from MSCs supernatant. **A** Representative TEM images showing cup-shaped morphology of MSC-EXOs. **B** Particle size distribution of MSC-EXOs was measured by NTA. **C** Representative plots of flow cytometry showing MSC-EXOs positive expression of the markers CD81 and CD9. **D** Western blot analysis showing the protein expression of CD9, CD81, TSG 101 and GM130

MSCs were firstly identified by flow cytometry with positive expression of MSC surface markers including CD29, CD44, CD90 and CD105, whereas negative for the expression of hematopoietic markers CD34 and CD45. Differentiation assays showed that MSCs could differentiate into adipocytes, osteocytes and chondrocytes under a conditioned medium (Additional file 1: Fig. S2). Then, EXOs purified from the supernatants of MSCs were identified. TEM revealed that EXOs exhibited a bilayer membrane structure with typical cup-shaped morphology (Fig. 1A). The majority of MSC-EXOs were in the size range from 30 to 150 nm (Fig. 1B). Meanwhile, exosome-specific markers CD9 and CD81 were confirmed by the flow cytometry (Fig. 1C). Using western blot analysis, exosome-specific markers including CD9, CD81 and TSG101 were identified in the exosome samples, whereas the non-exosomal marker GM130 was not detected (Fig. 1D).

### Exosomes mediated therapeutic effects of MSCs on liver fibrosis

**Fig. 2:**
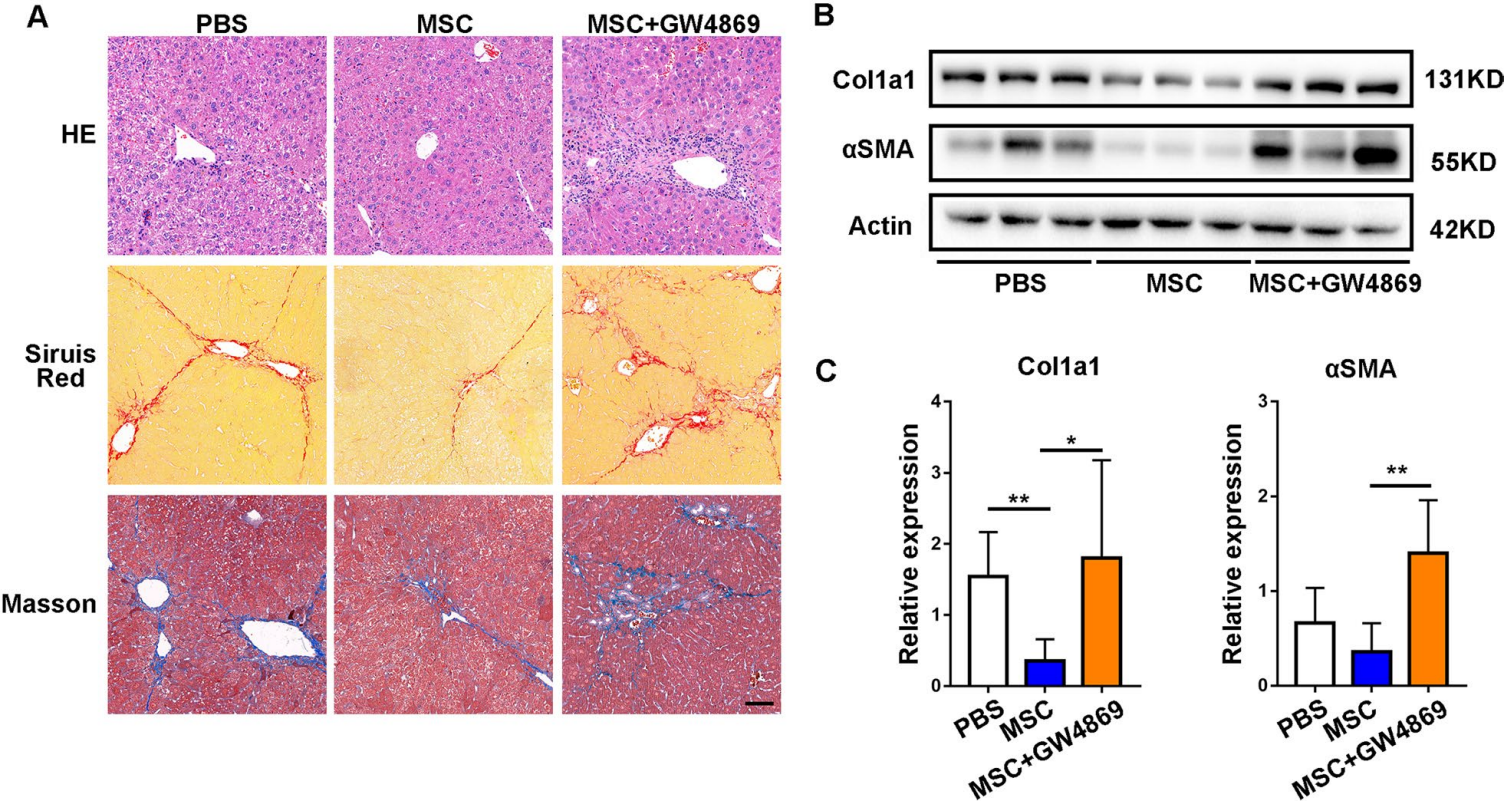
Blockade of exosomal generation of MSCs impaired the therapy for liver fibrosis. **A** Representative histological images of 2 weeks after PBS, MSC and MSC + GW4869 treatment. The top is HE staining, the middle is Sirius red staining and the bottom is Masson staining. Scale bars, 200 μm. **B** Western blot was used to measure the protein level of fibrotic Col1a1 and αSMA. **C** The mRNA levels of Col1a1 and αSMA were analyzed by RT-PCR assay

Increasing evidence demonstrates that therapeutic effects of MSCs largely depend on the paracrine effect [28]. Exosome is one of the most important mediators of MSCs in the paracrine activity. To verify the role of paracrine factors, particularly EXOs during MSCs treatment, we transplanted MSCs or GW4869 pre-treated MSCs into CCL4-induced mouse model of liver fibrosis. GW4869 is an inhibitor of neutral sphingomyelinase, which could be used to block exosome secretion [29]. The therapeutic effect was evaluated after two weeks by histopathology analysis. Compared with the PBS group, MSCs infusion demonstrated alleviation of inflammation and improvement of liver structure, while GW4869-pre-treated MSCs showed no obvious improvement of liver inflammation. Sirius red and Masson staining further showed that the application of GW4869 significantly inhibited the therapeutics effects of MSCs on CCL4-induced liver fibrosis; as a result, the transplanted MSCs failed to improve collagen deposition (Fig. 2A). The GW4869-dampened therapeutic effects were also confirmed by the elevated expression levels of Col1al and αSMA, as compared with the MSC-treated group (Fig. 2B, C).

**Fig. 3:**
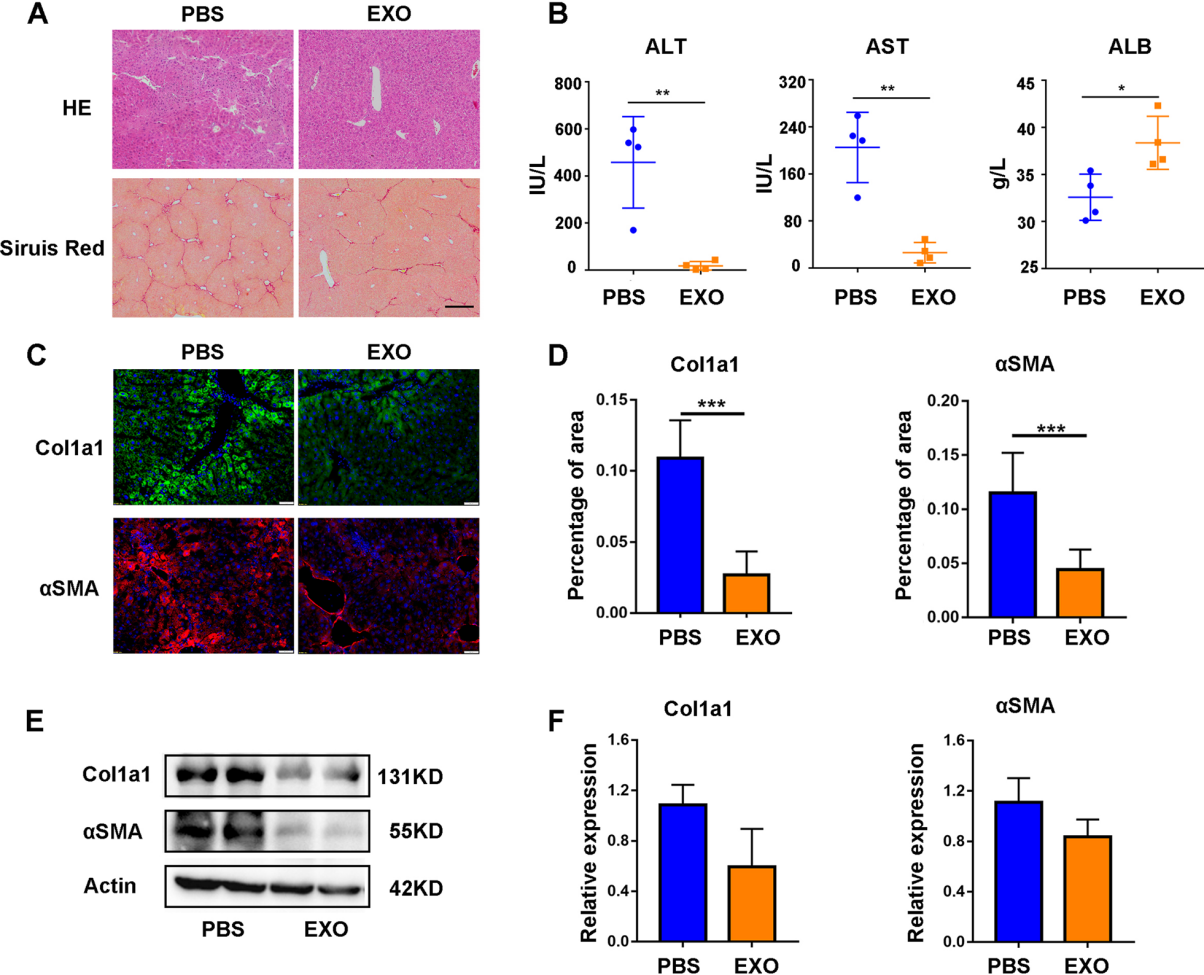
MSC-EXO treatment ameliorated liver fibrosis. **A** Representative histological images of PBS and EXO treatment by HE and Sirius red staining, 2 weeks after injection. **B** Changes of serum parameters including ALT, AST and ALB in different groups. **C** Representative images showed the Col1a1 + and αSMA + cells in each group. The nuclei were stained with DAPI. Scale bar, 50 μm. **D** Quantification of Col1a1 + and αSMA + areas by Image-Pro Plus. **E** The protein levels of Col1a1 and αSMA were measured by western blot. **F** The mRNA levels of Col1a1 and αSMA were measured by RT-PCR

To further evaluate the effects of EXOs, MSC-EXOs were isolated from supernatants of MSCs and injected into fibrotic mice via the tail vein. The exosome-treatment group displayed significant improvement in liver inflammation and fibrosis as assessed by H&E and Sirius red staining (Fig. 3A). Meanwhile, the serum ALT, AST, ALB tests indicated better hepatic function restoration by EXOs than PBS (Fig. 3B). Immunofluorescence analysis showed lower expression of Col1al and αSMA in the EXO group than in the control group (Fig. 3C, D). Besides, the expression levels of these fibrosis-related markers were also confirmed by WB and RT-PCR analysis (Fig. 3E, F). Collectively, these findings suggested that EXOs influence the therapeutic effect of MSCs on liver fibrosis.

### MSC-EXOs ameliorated the inflammatory response by the remodeling of macrophage phenotypes in vivo

**Fig. 4:**
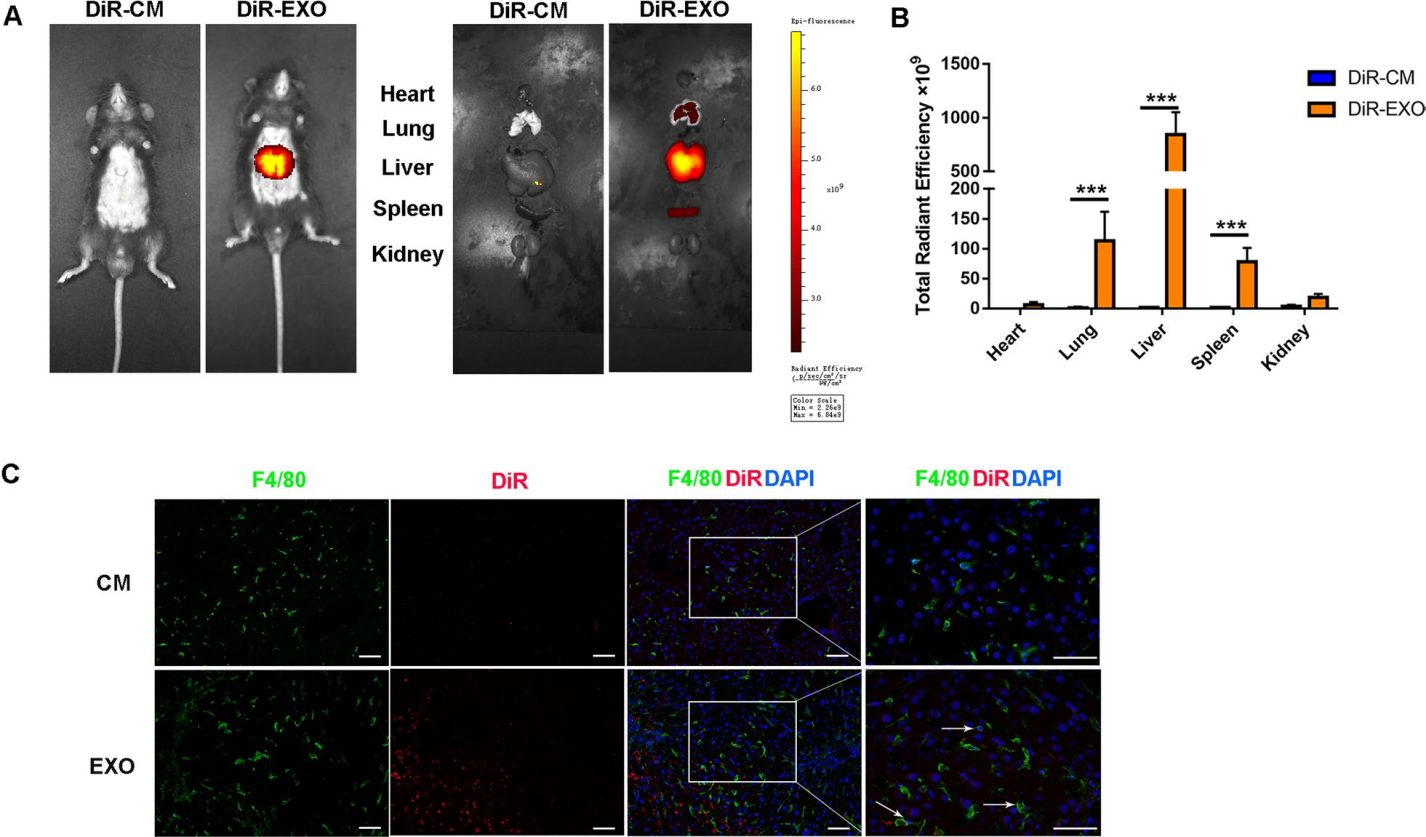
MSC-EXO circulated into the liver and co-located with macrophages. **A** Imaging of DiR-labeled exosomes from different tissues at 24 h after tail vein injection. **B** Quantification of the relative fluorescence intensity of the infiltrated exosomes in the tissues in different groups. **C** DiR-labeled exosome (red) localization in the liver as detected by fluorescence microscopy. Macrophages were F4/80 positive (green). Nuclei were counterstained with DAPI

Macrophages are key regulators of tissue fibrosis and execute an essential function in the initiation, maintenance and remodeling of tissue injury [30]. In addition, the polarization state of macrophage is closely associated with the development of liver disease [31]. To determine the macrophage polarization state in the progression of liver fibrosis, we tested M1 and M2 polarization markers by immunohistochemical staining. The results showed that the proportion of proinflammatory macrophage (iNOS^+^) increased significantly with the aggravation of hepatic fibrosis. Anti-inflammatory macrophages (CD206^+^) also exhibited an upward tendency, likely as a compensatory effect of inflammatory response (Additional file 1: Fig. S3). Previous studies have reported that tissue macrophage polarization is the key to the regulation of tissue fibrosis [32]. Therefore, we wondered whether MSC-EXOs exerted their biological functions by regulating the phenotype switching of macrophage. We detected the biodistribution of systematically delivered EXOs in the liver fibrosis model. As expected, exosomes were mainly distributed in the liver and spleen, and a small amount in lung (Fig. 4A, B), which was consistent with previous reports. Moreover, immunofluorescence staining analysis indicated that DiR-labeled EXOs were mainly localized in or near the F4/80-positive cells, suggesting that a cross-talk may exist between EXOs and macrophages (Fig. 4C).

**Fig. 5:**
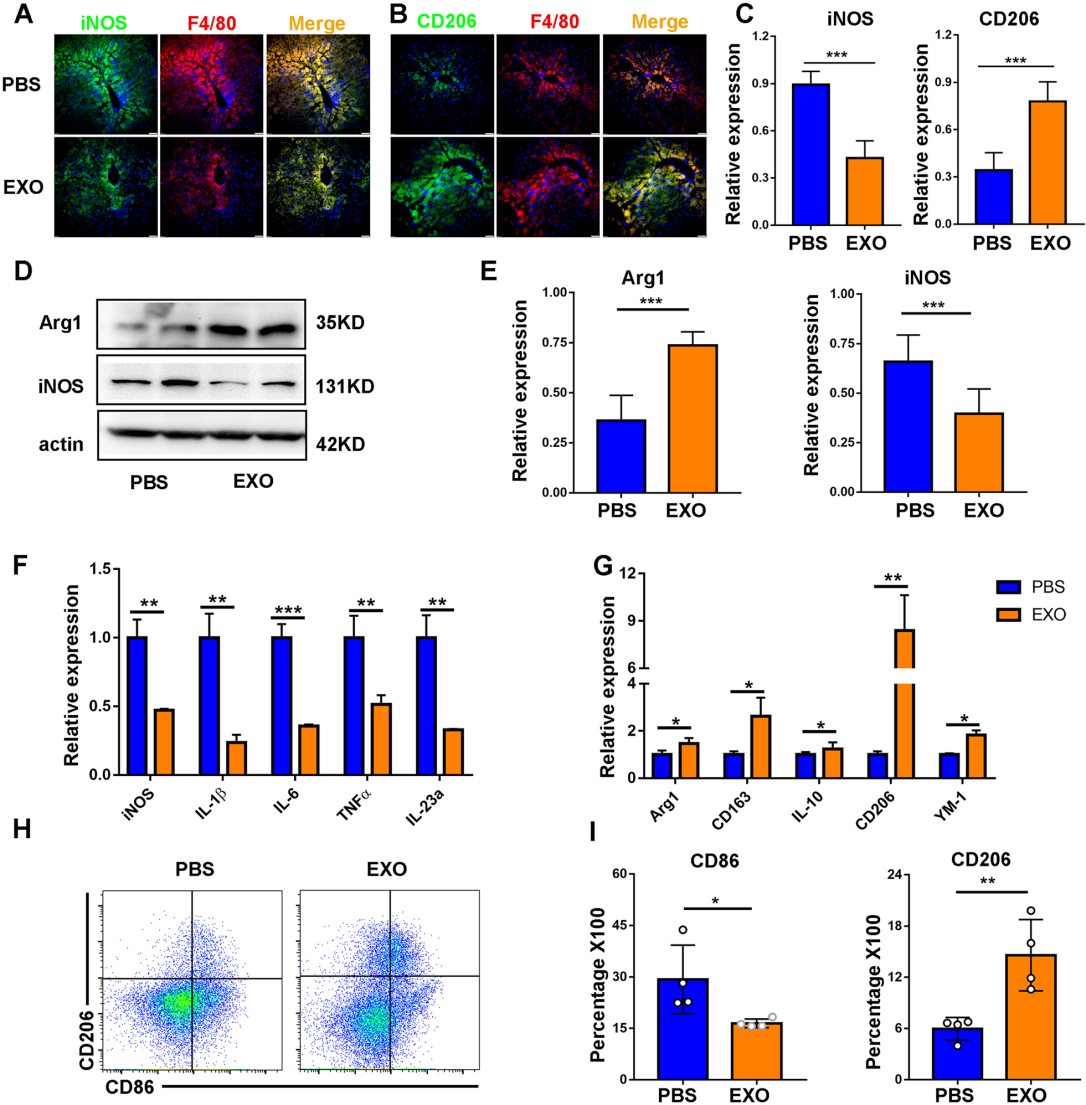
MSC-EXO inhibited pro-inflammatory macrophages and promoted anti-inflammatory macrophages in liver. **A**, **B** Representative images showed the iNOS + pro-inflammatory macrophages (green) and CD206 + anti-inflammatory macrophages (green) in each group. The nuclei were stained with DAPI (blue), and macrophages were stained with F4/80 (red). Scale bar, 100 μm. **C** The percentage of iNOS + or CD206 + cells was calculated by Image-Pro Plus. **D**, **E** The protein level of iNOS and Arg1 was measured by western blot, and quantification of the relative expression in the bands in different groups was calculated by Image-Pro Plus. **F**, **G** The mRNA levels of pro-inflammatory and anti-inflammatory macrophage markers were measured by RT-PCR. **H**, **I** Flow cytometry plots showing CD86 (M1 marker) and CD206 (M2 marker) changed in different groups and the percentage of CD86 + or CD206 + cells were analyzed by Flow Jo software

Based on these observations, we proceeded to investigate the regulatory effects of MSC-EXOs on macrophages. Liver sections were stained with anti-F4/80 antibody to identify infiltrated macrophages, and iNOS-positive cells were referred as M1 macrophages, while CD206-positive cells as M2 macrophages. Immunofluorescent staining showed that M1 macrophages (F4/80^+^iNOS^+^) significantly decreased due to MSC-EXO treatment compared with PBS. At the same time, M2 macrophages (F4/80 + CD206 +) showed an obvious increase in the EXO group (Fig. 5A–C). The expression of Arg-1 and iNOS in liver tissues was measured at protein levels by western blot. High expression of Arg-1 and low expression of iNOS were observed in the group receiving MSC-EXO-treated group (Fig. 5D and 5E). Consistently, the mRNA level of M1 markers (iNOS, IL-1β, IL-6, TNF-α and IL-23a) was downregulated, while the gene expression of M2 markers (Arg-1, CD163, IL-10, CD206 and Ym-1) was upregulated (Fig. 5F and G). Furthermore, flow cytometry analysis was used to quantify the proportion of M1 and M2 macrophages in the liver. As shown in Fig. 5H and I, the percentage of CD86-positive cells was decreased in the treatment group, whereas that of CD206-positive cells was significantly upregulated. These data suggested that the administration of MSC-EXOs contributed to the macrophage remodeling and exerted anti-inflammatory effects in the liver of mice with hepatic fibrosis.

### MSC-EXOs promoted the transition from M1 inflammatory phenotype to M2 anti-inflammatory phenotype in vitro

**Fig. 6:**
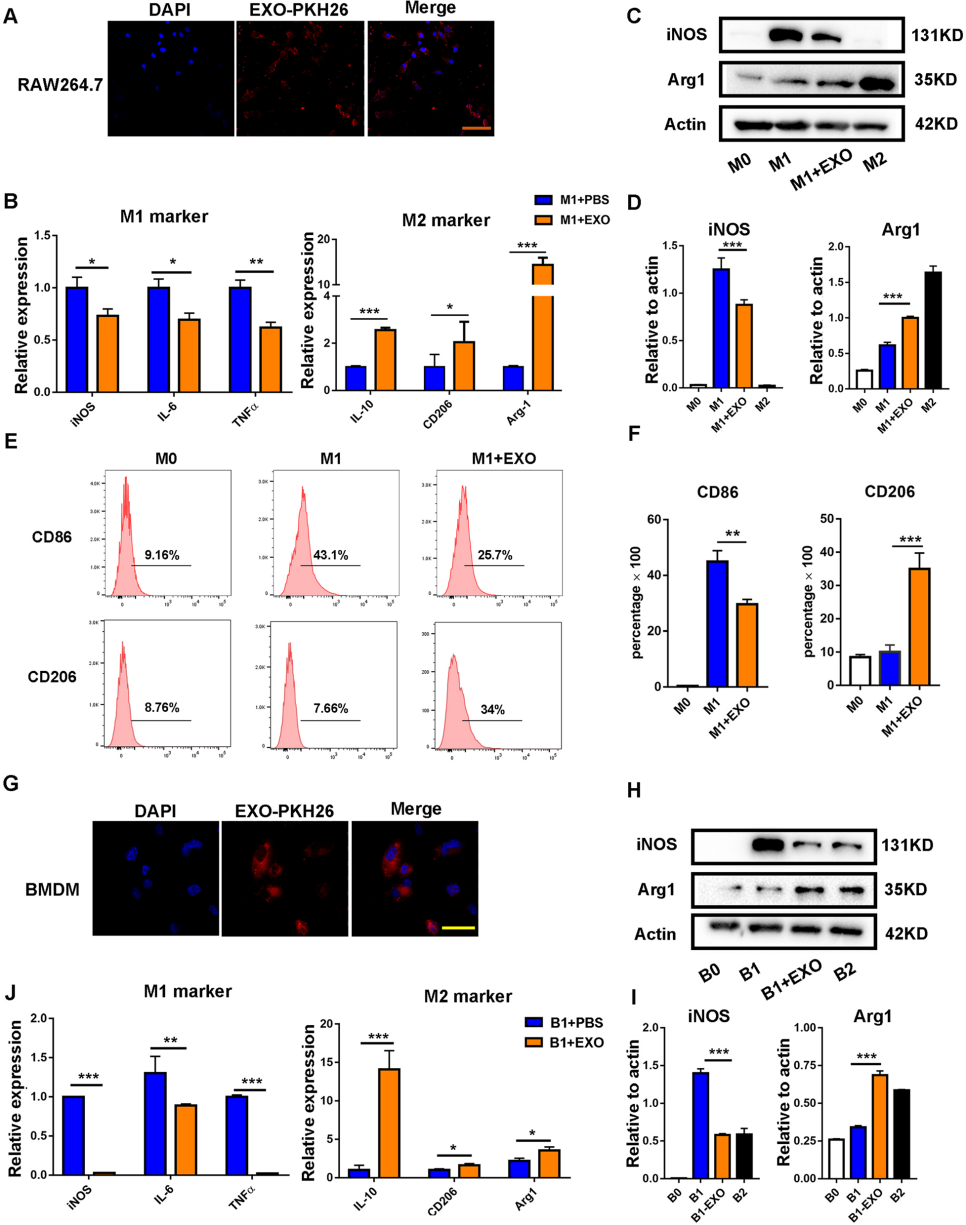
MSC-EXO induced the remodeling of macrophage phenotype in vitro. **A** Reparative confocal microscopy images showing colocalization of PKH26-labelled EXO with macrophage cell line RAW264.7. Scale bar = 50 μm. **B** RT-PCR analyzed the mRNA level of M1 and M2 markers of M1 + PBS (RAW264.7 + LPS/IFNγ + PBS) and M1 + EXO (RAW264.7 + LPS/IFNγ + EXO) groups. **C**–**D** The protein level of iNOS and Arg1 was measured by western blot, and quantification of the relative expression in the bands in different groups was calculated by Image-Pro Plus. **E**, **F** Reparative flow cytometry plots showing the percentage of CD86 (M1 marker) and CD206 (M2 marker) in different groups and the percentage of CD86 + or CD206 + cells was analyzed by GraphPad Prism (*n* = 4). **G** Reparative confocal microscopy images showing colocalization of PKH26-labelled EXO with BMDM. Scale bar = 20 μm. **H**, **I** The protein level of iNOS and Arg1 was measured by western blot and quantification of the relative expression in the bands in different groups was calculated by Image-Pro Plus. **J** RT-PCR analyzed the mRNA level of M1 and M2 markers of B1 + PBS (BMDM + LPS/IFNγ + PBS) and B1 + EXO (BMDM + LPS/IFNγ + EXO) groups

To further confirm the regulatory effects of MSC-EXOs on macrophages in vitro, PKH-26-labeled MSC-EXOs were co-cultured with RAW264.7 cells or BMDMs for 6 h. Immunofluorescence microscopy (Fig. 6A and G) showed the presence of EXOs (red) in the cytoplasm of macrophages stained with DAPI (blue), indicating that MSC-EXOs could be directly engulfed by macrophages. Next, RAW264.7 cells and BMDMs were treated with LPS/IFN-γ or IL-4 to acquire the polarization state toward the M1 or M2 phenotype and co-cultured with MSC-EXOs. We detected the expression of M1/M2 markers by RT-PCR and western blot analysis. The results showed that MSC-EXOs significantly decreased the level of M1 markers including iNOS, IL-6, TNF-α and increased the expression of IL-10, CD206 and Arg-1, which were representative markers of M2 macrophage (Fig. 6B–D). Meanwhile, the proportion of M1 macrophages expressing CD86 and M2 macrophages with positive expression of CD206 showed a consistent change tendency via flow cytometric analysis (Fig. 6E and F). Besides, similar findings were also verified by RT-PCR and western blot analysis in BMDM cells (Fig. 6H–J). Taken together, these results demonstrated MSC-EXOs inhibited inflammatory response by promoting M1 macrophage toward M2-like phenotype in vitro.

### miR-148a functioned as a critical effector in MSC-EXOs mediated macrophage polarization

**Fig. 7:**
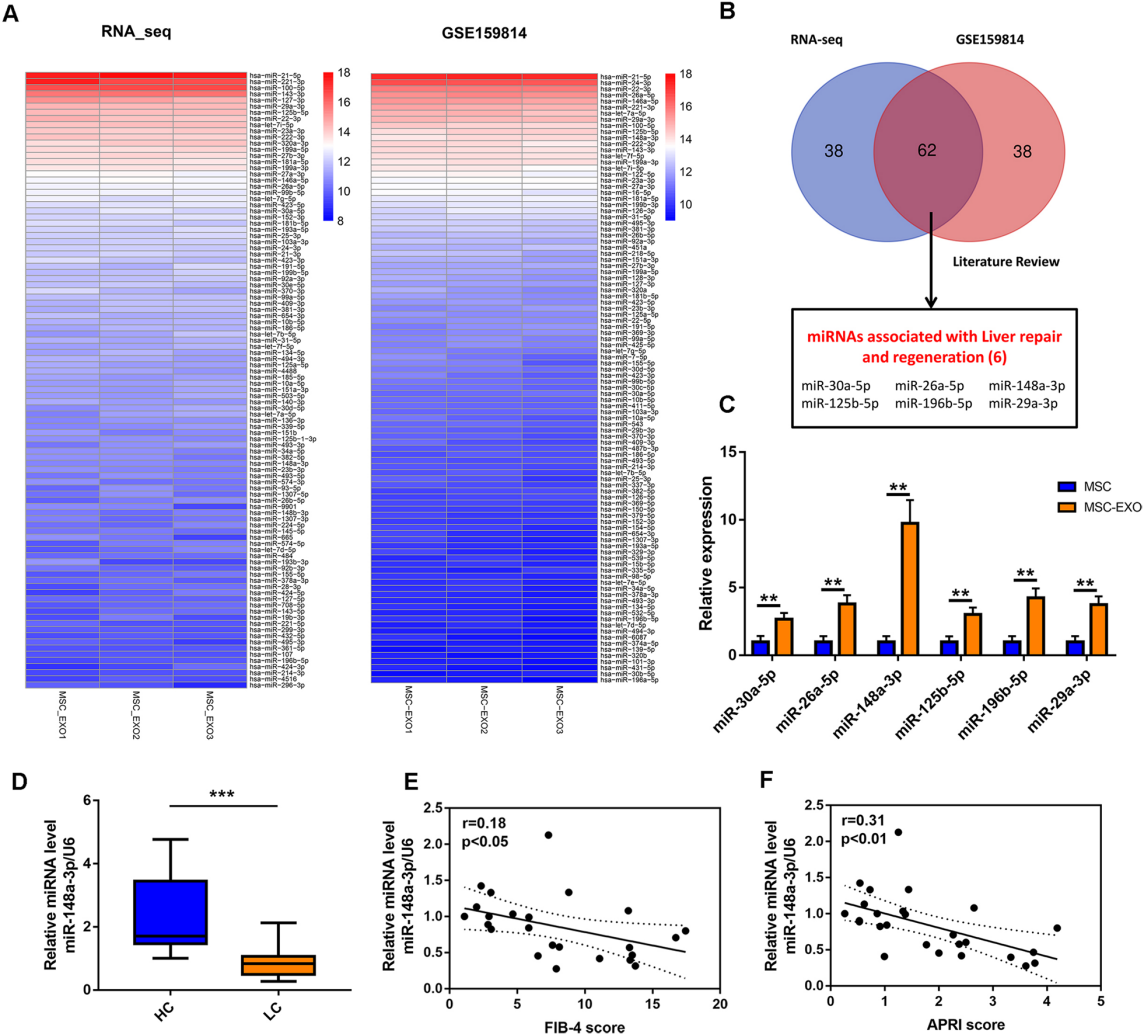
Exosomal miRNA profiling of MSCs and identification of miR-148a as an important content of MSC-EXO. **A** Heatmap showing the top 100 MSC exosomal miRNAs of RNA-seq and GSE159814 based on their expression value. **B** Selection of miRNAs related to liver repair and regeneration. **C** Comparison of six liver regeneration-related exosomal miRNAs: miR-30a-5p, 26a-5p, 148a-3p, 125b-5p, 196b-5p, and 29a-3p between MSC and MSC-EXO by RT-PCR. **D** Serum miR-148a mRNA levels in healthy controls and patients with liver cirrhosis were measured by RT-PCR and normalized using U6 (*n* = 24). **E**, **F** The correlation of serum miR-148a levels with FIB-4 and APRI score in patients with liver cirrhosis

EXOs are loaded with numerous bioactive molecules, especially miRNAs, which are involved in intercellular communication. To identify the cargos that might contribute to the therapeutic effects of MSC-EXOs, we performed small RNA sequencing and aligned these data with the GEO database (GSE 159814). Heatmap showed the top 100 miRNAs of the two sets of data based on their expression value, of which 62 miRNAs fall into the intersection (Fig. 7A, B). Through a systematic review of relevant literature, we identified six miRNAs (miR-30a-5p, miR-26a-5p, miR-148a-3p, miR-125b-5p, miR-196b-5p and miR-29a-3p) associated with the liver repair or regeneration. Expression level of these selected miRNAs was further validated by RT-PCR, and miR-148a was found to be the most abundant in the MSC-EXOs compared with MSCs (Fig. 7C). Furthermore, we investigated the expression of miR-148a in serum specimens from healthy donors and patients with liver fibrosis. As shown in Fig. 7D, patients with liver fibrosis showed lower serum miR-148a-3p level than healthy volunteers. Correlation analysis also showed that the serum miR-148a-3p was negatively correlated with the FIB-4 score and APRI score, reflecting the degree of liver fibrosis (Fig. 7E, F).

**Fig. 8:**
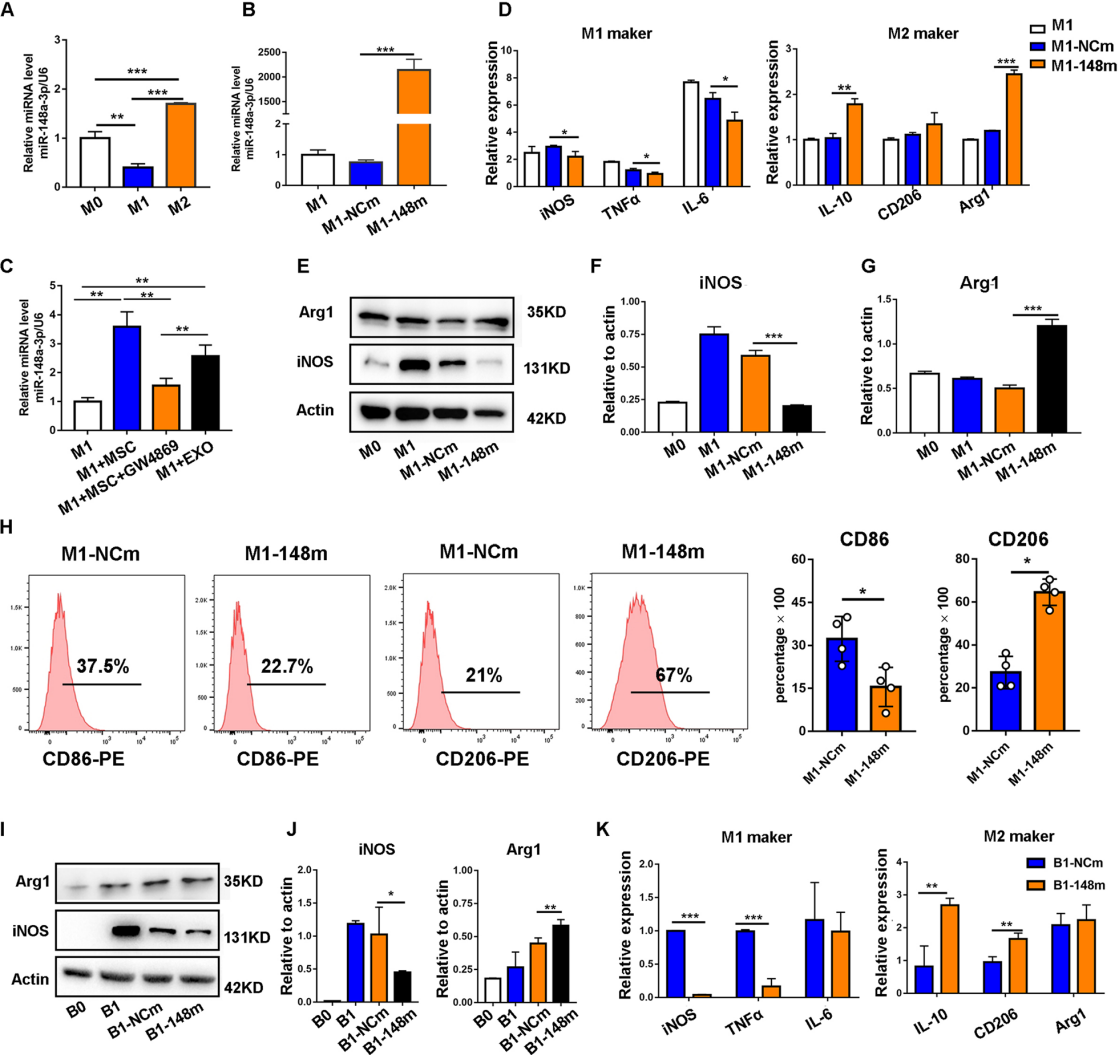
Overexpression of miR-148a suppressed M1 and promoted M2 macrophage polarization. **A** The miRNA level of miR-148a in different groups: M0 (RAW264.7), M1 (RAW264.7 + LPS/IFNγ) and M2 (RAW264.7 + IL-4) was measured by RT-PCR. **B**, **C** The mRNA level of miR-148a of each group was measured by RT-PCR. **D** RT-PCR was applied to measure the mRNA level pro-inflammatory and anti-inflammatory makers in RAW264.7 with miR-148a overexpression. **E**–**G** iNOS and Arg1 protein expression of RAW264.7 cell line with miR-148a overexpression were detected by Western blot. β-Actin protein levels were determined in parallel for loading control purposes. Densitometric analysis for the protein expression of iNOS and Arg1. **H** Reparative flow cytometry plots showing the percentage of CD86 (M1 marker) and CD206 (M2 marker) in different groups and the percentage of CD86 + or CD206 + cells were analyzed by GraphPad Prism (*n* = 4). **I**, **J** iNOS and Arg1 protein expression of BMDM with miR-148a overexpression were detected by Western blot. β-actin protein levels were determined in parallel for loading control purposes. Densitometric analysis for the protein expression of iNOS and Arg1. **K** RT-PCR was applied to measure the mRNA level pro-inflammatory and anti-inflammatory makers in BMDM with miR-148a overexpression

Next, we investigated the direct effects of miR-148a on macrophages. We found that the expression level of miR-148a was significantly reduced in M1 macrophages, but increased in M2 macrophages (Fig. 8A). The mimics were used to overexpress miR-148a-3p in RAW264.7 cells, and transfection effect was verified by RT-PCR (Fig. 8B). To prove that MSCs could deliver miR-148a to macrophages via EXOs, a co-culture system was established. In this system, M1 macrophages were cultured alone or co-cultured with MSCs, and the expression of miR-148a was measured by RT-PCR in macrophages. Macrophages co-cultured with MSCs had a high expression level of miR-148a, which could be decreased by the pretreatment of GW4869 on MSCs. In comparison, the expression level of miR-148a was also remarkably upregulated by the direct addition of MSC-EXOs (Fig. 8C). Then, we proceeded to explore whether miR-148a overexpression in M1 macrophages participated in the plasticity of macrophages by promoting the transition from M1 macrophages to M2 macrophages. In these experiments, the relative levels of M1 markers iNOS, TNF-α and IL-6 were significantly decreased in M1 macrophages transfected with miR-148a mimics compared with those transfected with control mimics. Meanwhile, miR-148a overexpression in M1 macrophages upregulated the expression of M2-polarized related markers including IL-10, CD206 and Arg-1 (Fig. 8D–G). In addition, a similar macrophage polarization pattern was shown in flow cytometric analyses, with a smaller proportion of M1 macrophages and a larger proportion of M2 macrophages in miR-148a mimics-transfected group than in the mimics control group (Fig. 8H). Similar results were also corroborated in primary BMDM cells (Fig. 8I–K).

Finally, to test whether miR-148a is essential for MSC-EXOs to regulate macrophage polarization, we treated M1 macrophages with MSC-EXOs or miR-148a inhibitor MSC-EXOs and then detected relevant markers of macrophage polarization. The results showed that MSC-EXOs treatment led to the downregulation of M1 markers (iNOS, TNF-α, IL-6) and upregulation of M2 markers (IL-10, CD206, Arg-1). However, these beneficial effects could be partially negated by miR-148a inhibitor MSC-EXOs (Additional file 1: Fig. S4). From these results, we could conclude that miR-148a, serving as an important effector in MSC-EXOs, plays a key role in the immunomodulation of macrophage phenotype.

### miR-148a directly targeted KLF6 to modulate macrophage polarization

**Fig. 9:**
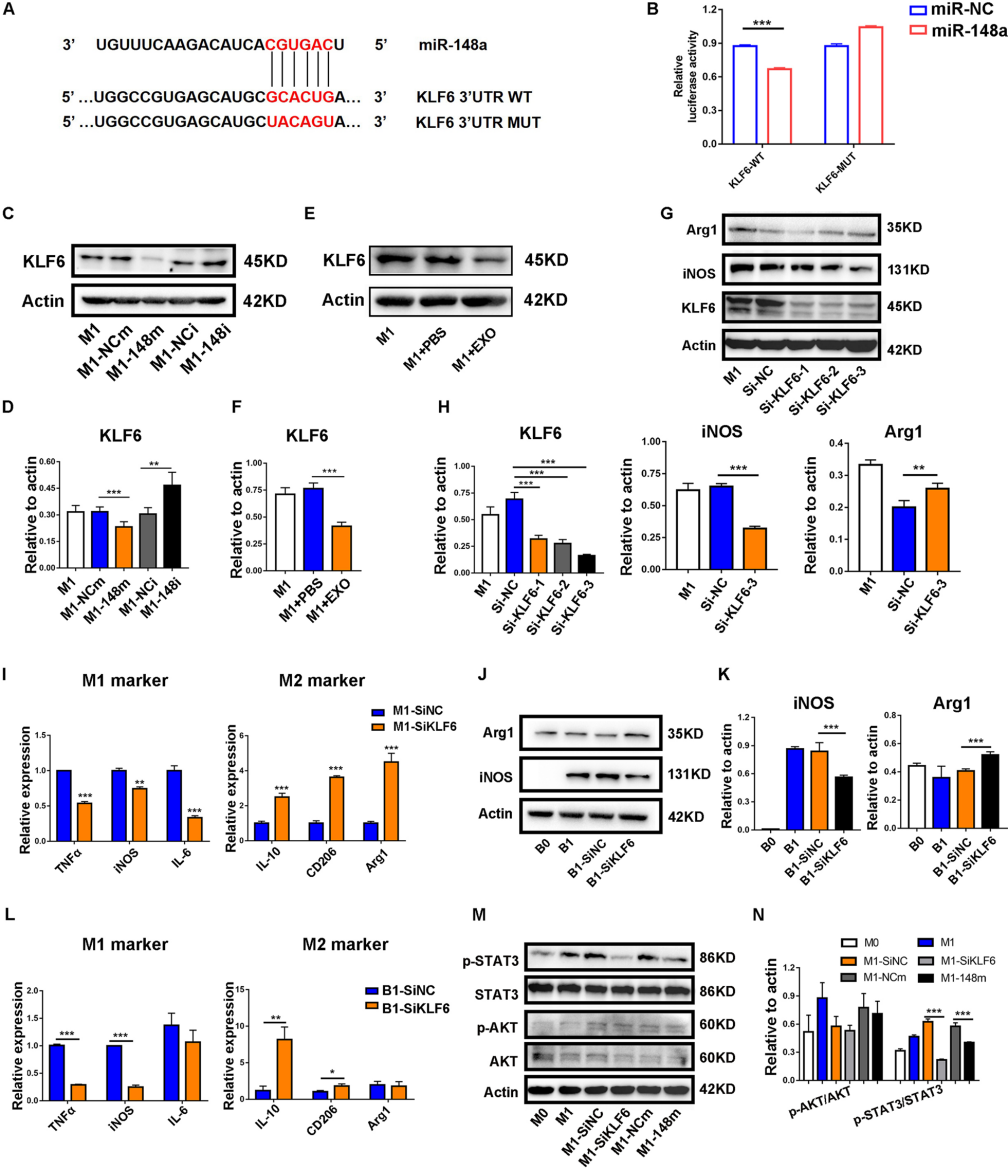
MiR-148a targetedly suppressed the expression of Kruppel-like factor 6 (KLF6). **A** The potential binding sites of human miR-148a on KLF6 3′UTR. **B** Luciferase reporter assay was performed to determine the interaction between miR-148a and KLF6. **C**–**F** The protein level of KLF6 and quantification of KLF6 were calculated by Image-Pro Plus. **G**, **H** The protein level of KLF6, iNOS and Arg1 was measured by western blot, and quantification of KLF6, iNOS and Arg1 was calculated by Image-Pro Plus. **I** RT-PCR was applied to measure the mRNA level pro-inflammatory and anti-inflammatory makers in RAW264.7 transfected with KLF6 siRNA. **J**, **K** The protein level of iNOS and Arg1 of BMDM was measured by western blot, and quantification of iNOS and Arg1 was calculated by Image-Pro Plus. **L** RT-PCR was applied to measure the mRNA level pro-inflammatory and anti-inflammatory makers in BMDM transfected with KLF6 siRNA. **M**, **N** Western blot was employed to measure the expression of pathway-related proteins. Quantification of p-AKT/AKT and p-STAT3/STAT3 was calculated by Image-Pro Plus

To explore the molecular mechanism by which miR-148a regulates the phenotypic switching of macrophages, we predicted the potential target genes of miR-148a through the miRanda, miRDB, TargetScan and CLIP database. Among all the candidates, KLF6 was reported to function as a transcriptional factor, promoting the inflammatory response in macrophages. To confirm the direct interaction between miR-148a and KLF6, we performed a luciferase reporter assay. The results showed that miR-148a mimics significantly reduce the luciferase activity in RAW 264.7 cells transfected with KLF6 wild-type sequence, but not the mutant sequence (Fig. 9A, B). Western blot analysis showed that KLF6 expression was markedly decreased with miR-148a mimics in RAW264.7 cells, whereas miR-148a inhibitor exhibited the opposite result (Fig. 9C and D). We also observed decreased KLF6 expression following the treatment of MSC-EXOs (Fig. 9E and F). Moreover, knocking down KLF6 by small interfering RNA resulted in the downregulation of M1 macrophage marker genes and the upregulation of M2 macrophage marker genes in both RAW264.7 and BMDM cells (Fig. 9G–K).

To further define the molecular mechanism underlying the induction of inflammatory response by KLF6, we mined a GEO dataset containing two sets of sequencing data of BMDMs from *Lyz2*^*cre*^ and *Klf6*^*fl/fl*^*:Lyz2*^*cre*^ mice. The heatmap showed the gene expression pattern of two groups (Additional file 1: Fig. S5A). GSEA and GSVA were conducted to reveal significant differential gene sets between two groups in response to inflammatory agent stimuli (Additional file 1: Fig. S5B and C). We then identified a total of 16 significantly enriched pathways commonly overlapping in both GSEA and GSVA (Additional file 1: Fig. S5D). Previous studies have reported that JAK/STAT3 and PI3K/AKT signaling pathways were associated with the macrophage polarization. So, the protein expression levels of p-STAT3 and p-AKT were detected by western blot in M1 macrophages. As shown in Fig. 9M, overexpression of miR-148a or knockdown of KLF6 reduced the expression of p-STAT3 but did not affect p-AKT. Together, these findings suggested that miR-148a modulated the polarization of macrophage by directly targeting KLF6 to regulate JAK/STAT3 signaling pathways.

### miR-148a agomir infusion attenuated liver fibrosis in mice

**Fig. 10:**
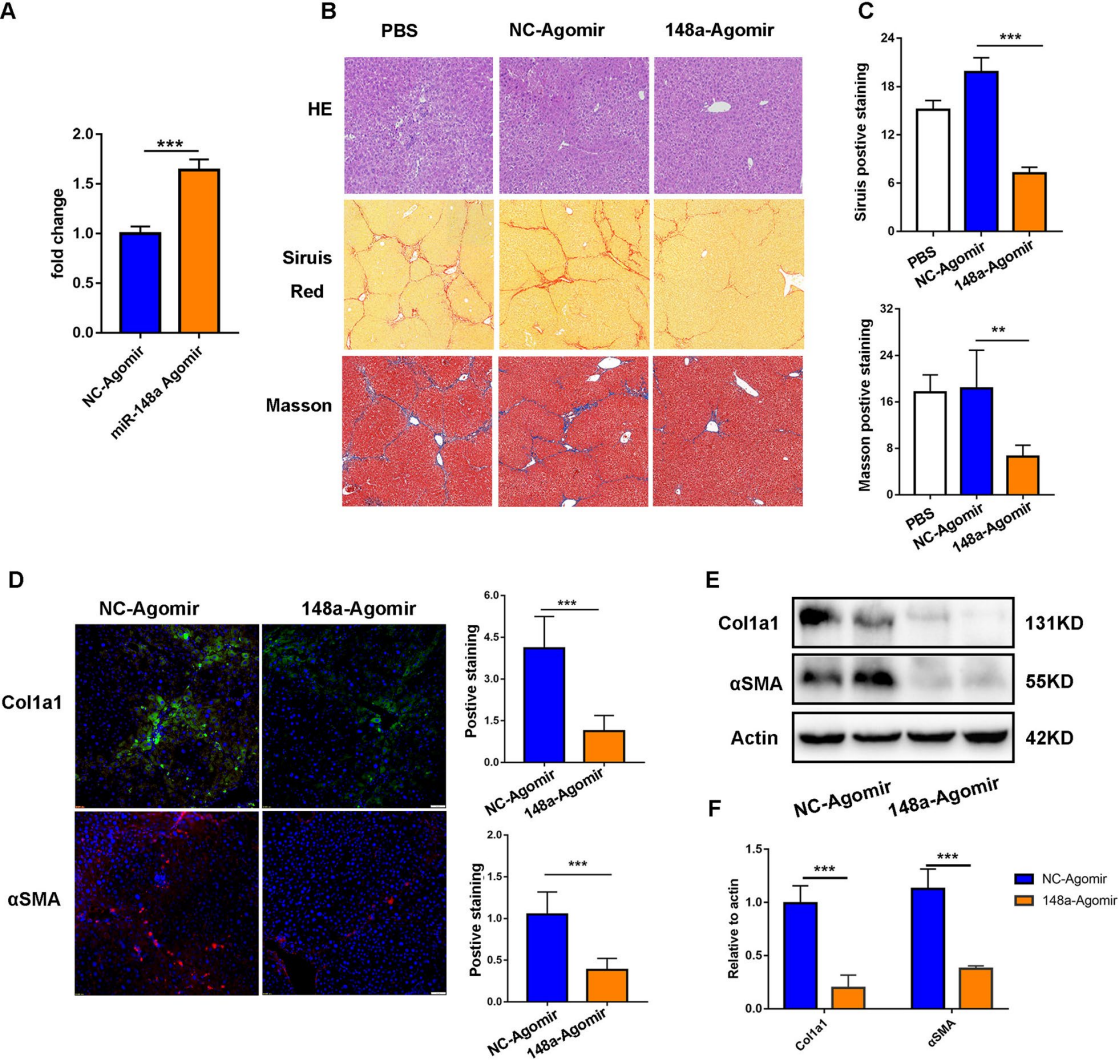
MiR-148a agomir infusion attenuated liver fibrosis in mice. **A** The level of miR-148a in liver was detected by RT-PCR assay. **B** Reparative histological images of PBS, NC-agomir and miR-148a agomir infusion by HE, Sirius red staining and Masson staining, two weeks after injection. **C** Quantification of fibrotic area (Sirius red and Masson). **D** Representative images showed the Col1a1 + and αSMA + cells in each group. The nuclei were stained with DAPI. Quantification of Col1a1 + and αSMA + areas was calculated by Image-Pro Plus. **E**, **F** The protein level of Col1a1, αSMA and quantification of Col1a1, αSMA bands related to actin was calculated by Image-Pro Plus

The above results strongly indicated that miR-148a might be a promising therapeutic target for liver fibrosis. We therefore sought to validate the therapeutic effect of miR-148a in vivo. Compared with MSC-EXOs, miR-148a-enriched MSC-EXOs significantly alleviated liver inflammation and fibrosis as indicated by histological analysis. Meanwhile, we also found that miR-148a knockdown diminished the therapeutic effects mediated by MSC-EXOs (Additional file 1: Fig. S6A). Western blot and RT-PCR experiments also confirmed these observations by the detection of fibrosis-related markers (Additional file 1: Fig. S6B–D). Next, we further evaluated therapeutic efficacy of miR-148a agomir in vivo. The systematic administration of miR-148a agomir markedly increased the expression of miR-148a in liver tissues of fibrotic mice (Fig. 10A). Consistent with MSC-EXOs treatment, miR-148a agomir infusion significantly attenuated liver fibrosis, which was evaluated by H&E, Sirius red staining and Masson staining (Fig. 10B and C). Furthermore, infusion of miR-148a also reduced the expression of hepatic fibrosis indicators including Col1al and αSMA (Fig. 10D–F). Meanwhile, the detection of the phosphorylation status of AKT and STAT3 also indicated that the activity of the STAT3 signaling pathway was repressed in the miR-148a agomir-treated group (Additional file 1: Fig. S7A, B). Above results confirmed the therapeutic effects of miR-148a and highlighted its potential clinical translational value.

## Discussion

Liver fibrosis is a result of chronic liver injury and ultimately leads to cirrhosis and end-stage liver failure. For patients with end-stage liver disease, liver transplantation is the only definitive treatment. However, lack of donors, postoperative complications and high cost limit its application in clinical practice. In recent years, results from clinical trials and animal models show that stem cell therapy holds exciting therapeutic promise for hepatic fibrosis [33, 34]. Nevertheless, the precise mechanism is not exact clear and remains to be further elucidated. In this study, we discovered MSCs transplantation ameliorated liver fibrosis in mice, which was largely mediated by EXOs via transferring their components to macrophages for intercellular communication. We further confirmed miR-148a was the critical effector in MSC-EXO-mediated therapeutic effects. Mechanistically, miR-148a directly targets KLF6 to modulate the polarization of macrophages via JAK/STAT3 pathways. Our findings confirmed EXOs as the potent paracrine mediators of MSCs in anti-fibrotic effects and proposed miR-148a as a potential therapeutic target for liver fibrosis.

Currently, MSCs have been one of the major hot topics in the field of regenerative medicine owing to their self-renewable capacities and multipotential differentiation. Previous studies have reported that MSCs could exert therapeutic effects by replacing damaged hepatocytes through transdifferentiation in vivo [35]. Promoting the hepatic differentiation of MSCs may contribute to the repair of liver injuries. However, increasing evidence indicated the efficiency of MSCs transplantation largely depended on the tissue microenvironment during the liver injury, which may be the key determinant of treatment efficacy [36]. MSCs, also known as “drug store”, could secret a variety of soluble factors including growth factors, chemokine and cytokines [37]. We previously reported that MSC released TSG-6 in a paracrine manner, participating in the remodeling of macrophage phenotype and promoting collagen fibers degradation [9]. In addition to these mediators above, EXOs have generated tremendous interest as the paracrine signals. Several studies have reported that MSC-EXOs are as effective as their parental cells in promoting liver repair or regeneration, by alleviating liver inflammation, inhibiting the activation of hepatic stellate cells and reducing the collagen deposition [38, 39]. Nevertheless, the studies of how MSC-EXOs affect the immune microenvironment during liver fibrosis are limited. In the present study, we found both MSCs and MSC-EXOs could ameliorate liver fibrosis in vivo. Notably, however, the efficacy of MSC treatment was significantly impaired by blocking exosome generation, demonstrating the indispensable role of EXOs in attenuating liver fibrosis. Further experiments confirmed the injected MSC-EXOs were primarily distributed in the liver and taken up by the macrophages. The clues suggested a possible communication between macrophages in the hepatic microenvironment and EXOs released by MSCs. Thus, in the following experiments, we focused on the interaction between MSC-EXOs and macrophages and sought to define the regulatory mechanism.

The activation of HSC is a pivotal event of liver fibrosis, but immune cells, especially macrophages, are the key modulators of HSC activation [31]. Duffield JS et al. [40] reported that removal of mouse macrophages during the progressive phase of liver fibrosis reduced the formation of liver scar, whereas depletion of macrophages during the recovery phase inhibited tissue repair. Macrophages are critically involved in the progression of liver injures and targeting macrophages represents an emerging anti-fibrotic therapeutic strategy. Furthermore, it’s widely accepted that macrophages are highly plastic and functionally divided into two major subsets, classically activated macrophages (M1) and alternatively activated macrophages (M2). M1 macrophages release proinflammatory cytokines to promote inflammation and exacerbate liver injuries. By contrast, M2 macrophages exert immunosuppressive effects and regulate tissue remodeling and repair [41]. Our work confirmed that MSC-EXOs effectively converted the polarization state of macrophages from M1 to M2 phenotype, not only in vitro but also in liver fibrosis models.

miRNAs have been implicated as one of the most abundant cargos of EXOs and largely determine the biological functions on their target cells. Phinney et al. [42] reported that EXOs derived from DicerKO MSCs had no significant effects on the macrophage activation, indicating the essential role of miRNAs in the MSC-EXOs. To identify exosomal miRNAs responsible for the biological effects, we performed RNA-sequencing of MSC-EXOs and aligned the data to the public database. We found miR-148a was highly expressed and acted as a critical effector in MSC-EXO-mediated macrophage polarization. Regarding the regulation of miR-148a on macrophages, Zheng et al. [43] reported that miR-148a overexpression could target CaMKII to inhibit inflammation of liver Kupffer cells during liver ischemia–reperfusion injury. It has also been suggested that miR-148a promotes the polarization of tumor macrophages to M2 phenotype and thus inhibits the migration of tumor cells [44]. Our previous studies unveiled miR-148a as an important molecule promoting the differentiation of MSCs into hepatocytes [18]. Here, in this study, we found miR-148a was poorly expressed in mouse models of liver fibrosis but markedly upregulated after MSC-EXOs treatment (data not shown). Furthermore, the injection of MSC-EXOs with miR-148a overexpression significantly attenuated liver fibrosis in mice. However, it is worth noting that miR-148a is just one component of MSC-EXOs. The treatment of MSC-EXOs might amplify the biological effects of miR-148a. To test the actual therapeutic effects of miR-148a, we also applied miR-148a agomir injection in vivo studies. The therapeutic outcomes were also satisfactory. All these observations suggested that miR-148a might be a promising therapeutic target. We also identified the potential mechanism underlying miR-148a-mediated macrophage polarization by modulating KLF6.

KLF6 is a zinc finger DNA-binding transcription factor that regulates gene expression, belonging to zinc finger domain family [45]. Zhang et al. [46] reported that KLF6 could act as NF-κB co-activator to promote the transcription of its downstream genes in renal tubular epithelial cells. Moreover, KLF6 has been shown to regulate macrophage functions by promoting inflammatory gene expression [47]. Overexpression of KLF6 led to the promotion of miR-223 expression, enhancing the proinflammatory macrophage activation [48]. There is also evidence suggesting that KLF6 facilitates the inflammatory response in macrophages by suppressing the PPARγ pathways [49]. In our study, KLF6 was predicted as the target gene of miR-148a and verified by a luciferase reporter assay. We also confirmed targeting KLF6 could modulate the phenotype switch of macrophages. Furthermore, results from GSEA and GSVA analysis identified two signaling pathways associated with macrophage polarization [31]. Following verification, we finally came to the conclusion that KLF6 modulated the polarization of macrophage through JAK/STAT3 pathways. However, the specific regulatory mechanisms require further investigation.

## Conclusion

**Fig. 11:**
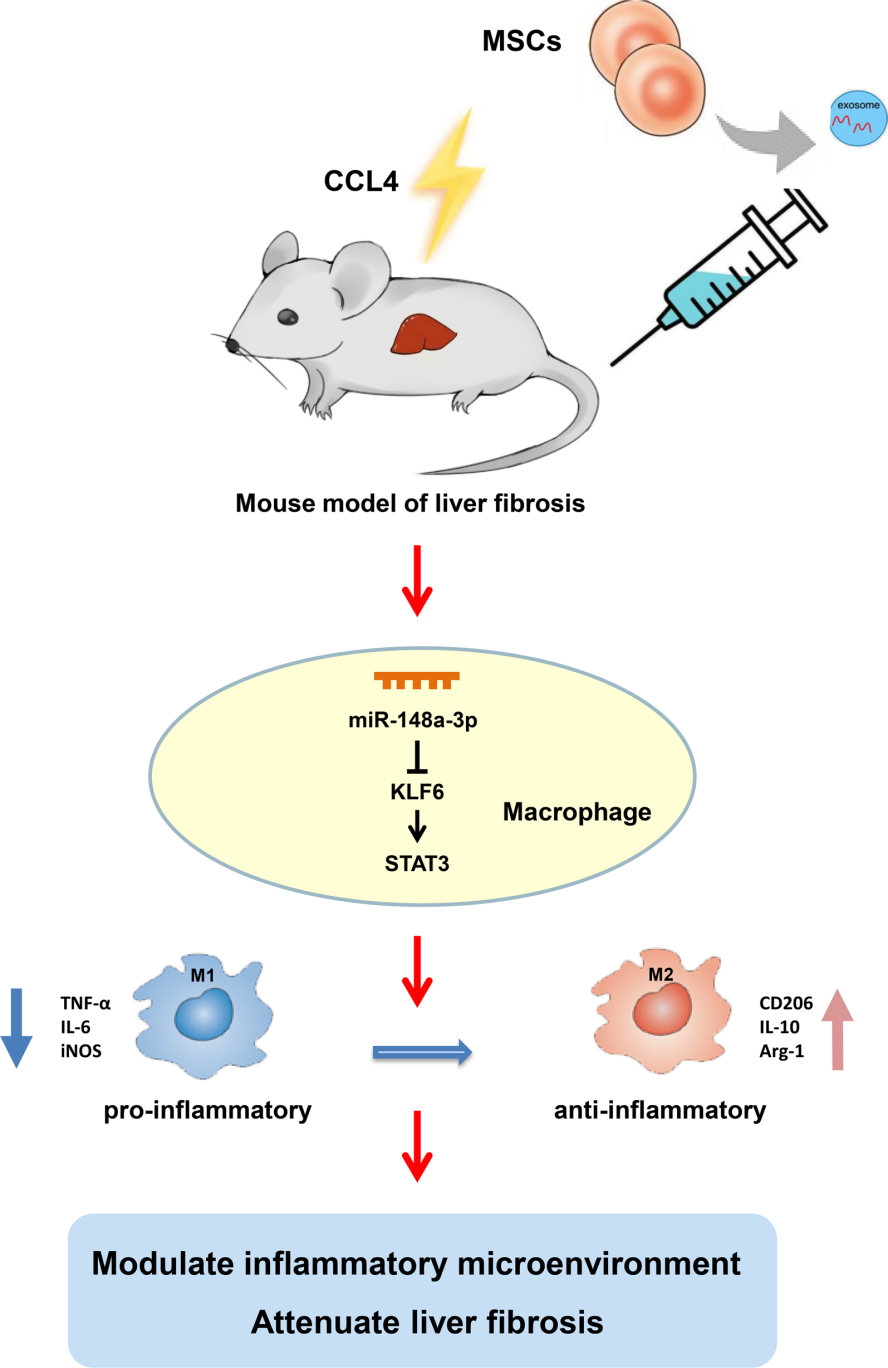
Schematic diagram of MSC-EXOs to attenuate liver fibrosis in the fibrotic mice model. Liver fibrosis can be ameliorated by the infusion of MSC-EXOs, which deliver miR-148a to intrahepatic macrophages targeting KLF6 to inhibit the STAT3 signaling pathway

In summary, we confirmed that MSC-EXOs ameliorate liver fibrosis by inducing proinflammatory macrophages into an anti-inflammatory phenotype. MiR-148a is identified as the therapeutic effector of MSC-EXOs. Mechanistically, miR-148a regulates the STAT3 signaling pathway by directly targeting KLF6 (Fig. 11). Our findings indicate that miR-148a might be a promising therapeutic target for the treatment of liver fibrosis.

## Supplementary Information

**Additional file 1**. Supplementary figures and tables.

## Data Availability

The datasets used and/or analyzed during the current study are available from the corresponding author on reasonable request.

## Abbreviations

MSC: Mesenchymal stem cells
MSC-EXOs: MSC-derived exosomes
KLF6: Kruppel-like factor 6
ECM: Extracellular matrix
MSC-CM: MSC-conditioned medium
MFGE8: Milk Fat Globule-EGF Factor 8
TSG-6: Tumor necrosis factor-α stimulated gene 6
MMP12: Metalloproteinase 12
IDO: Indoleamine-2,3-dioxygenase
PGE2: Prostaglandin E2
HLA-G: Human leukocyte antigen
CCL4: Carbon tetrachloride
STAT3: Signal transducer and activator of transcription 3
AASLD: American Association for the Study of Liver Disease
hUC-MSCs: Human umbilical cord-derived MSCs
GEO: Gene Expression Omnibus
GSEA: Gene set enrichment analysis
GSVA: Gene set variation analysis
