# Supplementary material for: Mesenchymal stem cell-derived exosomes protect against liver fibrosis via delivering miR-148a to target KLF6/STAT3 pathway in macrophages
Source: Stem Cell Res Ther. 2022 Jul 20;13:330. doi: 10.1186/s13287-022-03010-y (PMC9297598; doi:10.1186/s13287-022-03010-y)
Supplement: Supplementary file 1 — Additional file 1. Supplementary figures and tables. [file 13287_2022_3010_MOESM1_ESM.docx]

**Additional file 1: Fig. 1. Gating strategies for macrophages in the liver.**


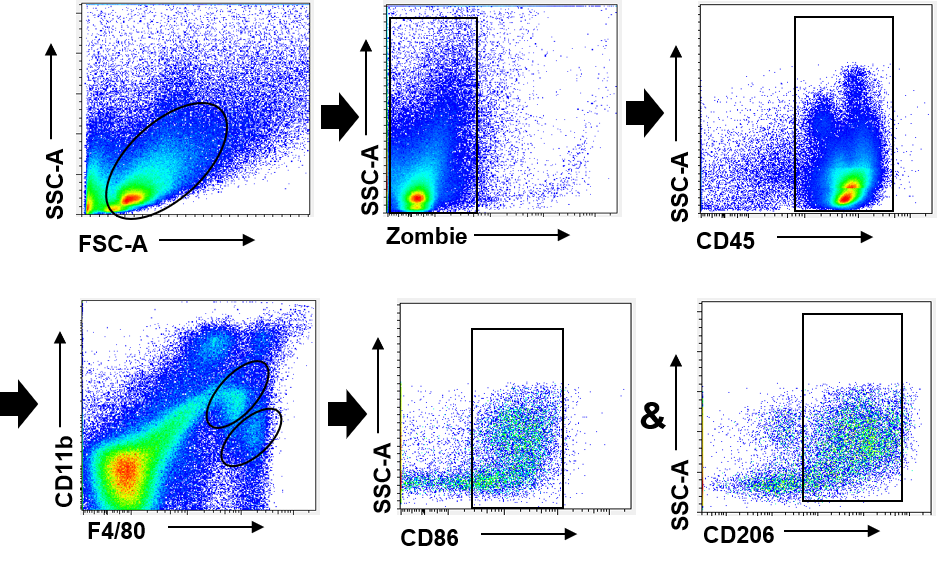


**Additional file 1: Fig. 2.** **Characterization of Mesenchymal stem cell（MSCs）derived from human umbilical cord.** A) Representative plots of flow cytometry showing MSCs positive expression of the surface markers CD105, CD29, CD90 and CD44 but negative of CD45 and CD34. B-D) Multipotential capabilities of MSCs to differentiate into osteoblasts, adipocytes, and chondrocytes by alizarin red staining (B), oil red O staining (C), and alcian blue staining (D).


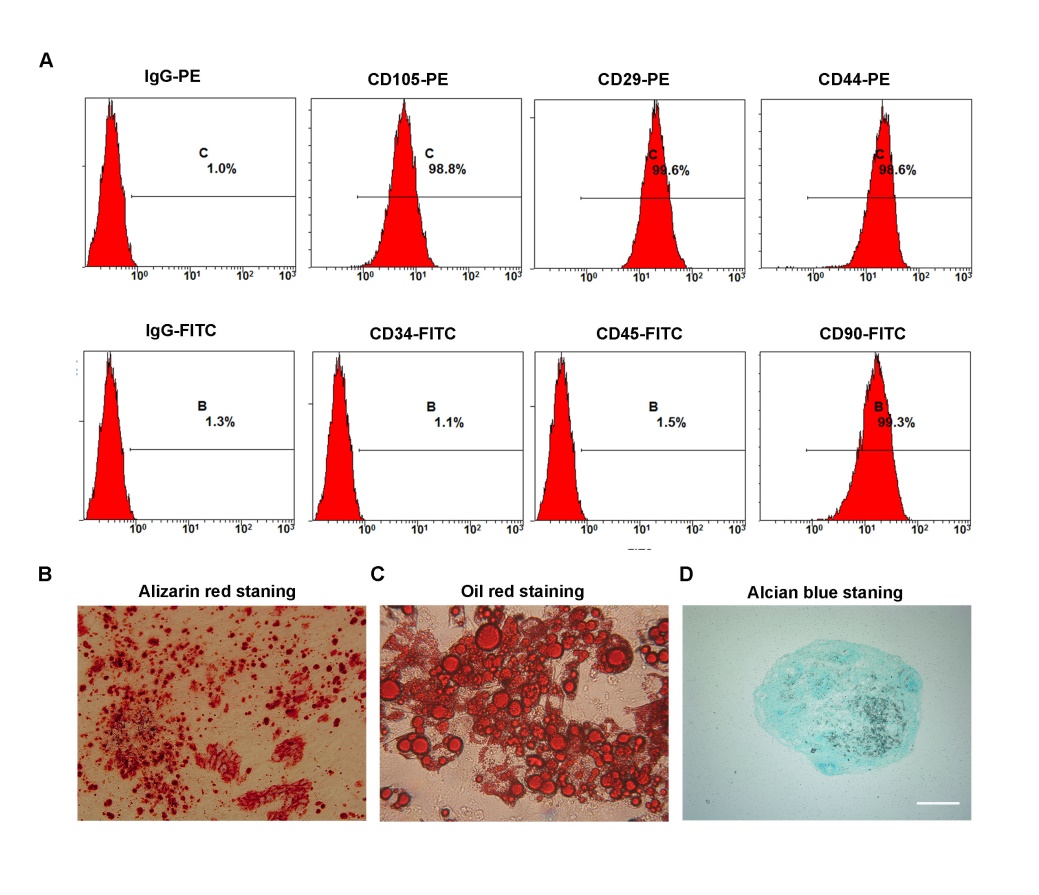


**Additional file 1: Fig. 3.** **Macrophage phenotype was changed during the progression of liver fibrosis induced by CCl4.** A) Representative liver histological images of 0, 2, 4, 6 and 8 weeks with HE, Sirius red and Masson staining. B) Expression of iNOS+ (M1 macrophage marker) and CD206+ (M2 macrophage marker) of different groups was analyzed by Immunohistochemical staining. C) Quantification of iNOS+ and CD206+ areas by Image-Pro Plus.


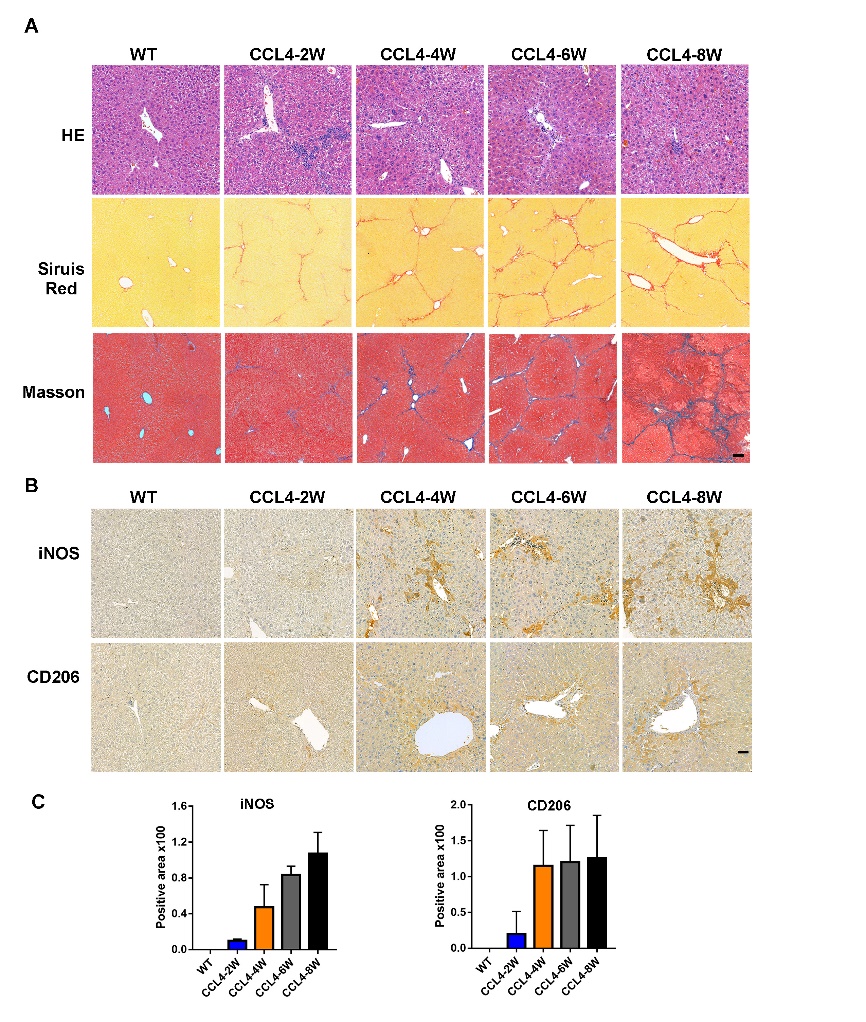


**Additional file 1: Fig. 4.** **Knockdown of miR-148a in EXO impaired the repression on pro-inflammatory macrophages.** A) RT-PCR was used to measure the expression of miR-148a. B) Expression of M1 and M2 macrophages markers of different groups was analyzed by RT-PCR. C-D) Protein expressions of iNOS and Arg1 were measured by western blot and quantification of iNOS and Arg1 bands were calculated by Image-Pro Plus.

12


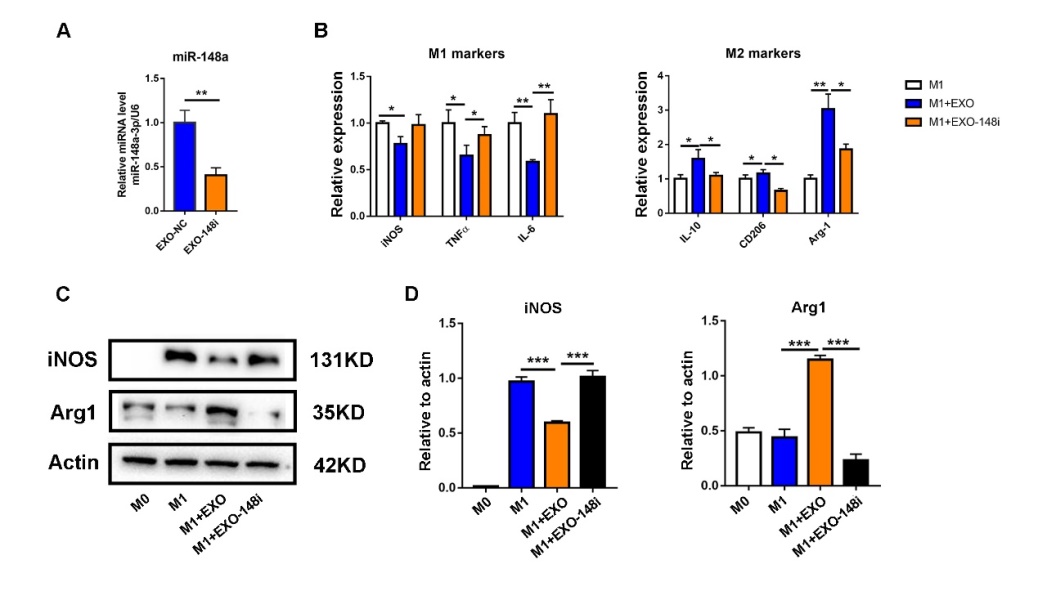


**Additional file 1: Fig. 5. GEO database (GSE13476) was employed to mine the potential signaling pathways underlying the induction of inflammatory response by KLF6.** A) Heatmap demonstrated the gene expression pattern of BMDMs from *Lyz2^cre^* and *Klf6^fl/fl^:Lyz2^cre^* mice. B-C) Bar plots showed the results of GSEA and GSVA. Red and blue bars were significant terms (P<0.05), representing up-regulation and down-regulation, respectively. D) Significant enriched pathways commonly overlapping in both GSEA and GSVA analysis.


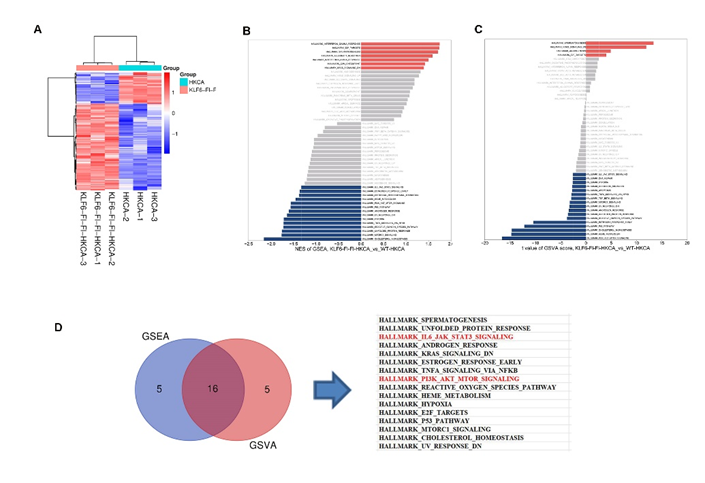


**Additional file 1: Fig. 6.** **MiR-148a-enriched MSC-EXOs enhanced the therapy for liver fibrosis.**  A) Reparative histological images of PBS, EXO, EXO-148m and EXO-148i treatment, two weeks after injection. B) The mRNA levels of Col1a1 and αSMA in liver tissues were measured by RT-PCR assay. C-D) The protein level of Col1a1, αSMA and quantification of Col1a1, αSMA bands related to actin was calculated by Image-Pro Plus.


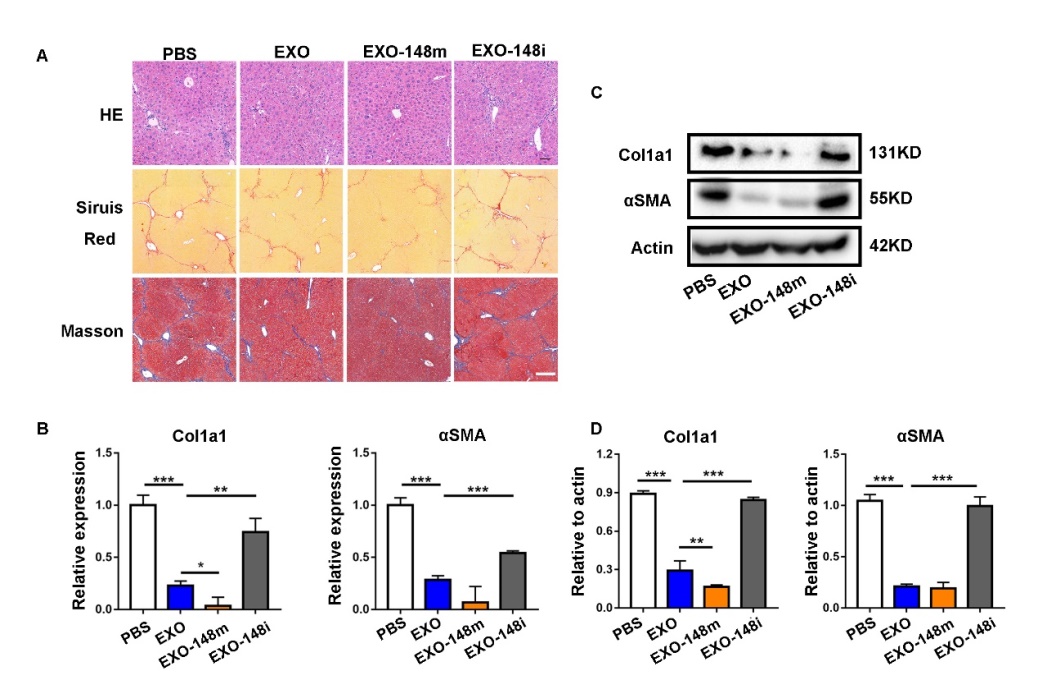


**Additional file 1: Fig. 7.** **MiR-148a inhibited the KLF6 expression and STAT3 signaling pathway in mice fibrotic livers.** A) Protein expression of P-STAT3, STAT3, P-AKT, AKT and KLF6 were detected by western blot. B) Quantification of the relative expression in the bands in different groups was calculated by Image-Pro Plus.

**
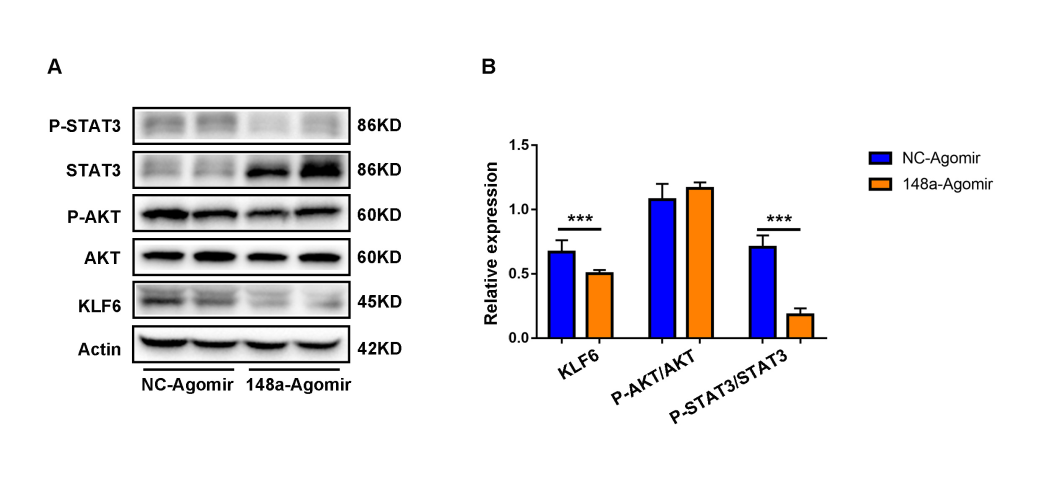
**

**Additional file 1: Table 1. The detail information of healthy volunteers and patients involved in this study.**

| **Characteristic** | **Healthy volunteers (n=12)** | **Cirrhosis patients (n=24)** | ***P* value** |
| --- | --- | --- | --- |
|  |  |  |  |
| **Age (year)** | 48.33 ± 7.34 | 54.08 ± 9.15 | 0.067 |
| **Female (%)** |  |  |  |
| **PLT (× 109/L)** | 243 (212.25, 277) | 53.5 (33.75, 93) | < 0.001 |
| **ALT (IU/L)** | 18.5 (13.75, 22.75) | 24.5 (20, 33.75) | 0.009 |
| **AST (IU/L)** | 18 (16.75, 21) | 33 (27, 43.5) | < 0.001 |
| **ALB (g/L)** | 45.42 ± 2.02 | 42.01 ± 5.87 | 0.015 |
| **TBIL (μmol/L)** | 12 (10.88, 15.82) | 23.2 (16.15, 32.35) | < 0.001 |
| **Fibrosis score** |  |  |  |
| **FIB-4** | — | 1.9 ± 1.18 |  |
| **APRI** | — | 8.01 ± 4.94 |  |

Data are expressed as the Median (25th, 75th percentiles) or N (%). Abbreviations: n, number of subjects; ALT, alanine aminotransferase; AST, aspartate aminotransferase; TBIL, total bilirubin; ALB, albumin. Student’s t test or Wilcoxon rank-sum test for continuous factors and Fisher's exact test for categorical factors were used.

**Additional file 1: Table 2. The information of antibodies used for flow cytometric.**

| **Antibody** | **Company** | **Catalog Number** |
| --- | --- | --- |
| **CD45** | Biolegend | 109824 |
| **CD11b** | Biolegend | 101206 |
| **F4/80** | Biolegend | 123110 |
| **CD86** | Biolegend | 105028/159204 |
| **CD206** | Biolegend | 141717/141706 |
| **Zombie** | Biolegend | 423113 |

**Additional file 1: Table 3. Primers used in the RT-PCR analysis.**

| **Gene** | **Forward Primer** | **Reverse Primer** |
| --- | --- | --- |
| **Actin** | CAGCACAATGAAGATCAAGATC | CGGACTCATCGTACTCCTGCTT |
| **Col1a1** | TCTAGACATGTTCAGCTTTGTGGAC | TCTGTACGCAGGTGATTGGTG |
| **αSMA** | CGAGCCGAGAGTAGCAGTTGTAG | AGCCATTGTCGCACACGAG |
| **iNOS** | AGTCTCAGACATGGCTTGCCCCT | GCTGCGGGGAGCCATTTTGGT |
| **CD206** | CGAGCCGAGAGTAGCAGTTGTAG | AGCCATTGTCGCACACGAG |
| **Arg1** | CTCCAACCCAAAGACCTTAGTG | AGGAGCAGTCATTCGGGACTTC |
| **TNFα** | GACCCTCACACTCAGATCATCT | CCTCCACTTGGTGGTTTGCT |
| **IL-6** | CCACTTCACAAGTCGGAGGCTTA | GCAAGTGCATCATCGTTGTTCATAC |
| **IL-10** | ACTCTTCACCTGCTCCACTG | GCTATGCTGCCTGCTCTTAC |
| **IL-23a** | AGGACGTGTGTTGTTATTGTTCTGT | CTCTGGCGTTTGTTTCTTTTATCTT |
| **YM-1** | AGAAGGGAGTTTCAAACCTGGT | GTCTTGCTCATGTGTGTAAGTGA |
| **IL-1β** | TCCAGGATGAGGACATGAGCAC | GAACGTCACACACCAGCAGGTTA |
| **CD163** | ACTTCACAATCACTTCATGACACA | TCGTCGATTCAGACTCCTCAG |
| **hsa-miR-148a-3p** | TCAGTGCACTACAGAACTTTGT |  |
| **hsa-miR-26a-5p** | TTCAAGTAATCCAGGATAGGCT |  |
| **hsa-miR-30a-5p** | CGTGTAAACATCCTCGACTGGAAG |  |
| **hsa-miR-196b-5p** | TAGGTAGTTTCCTGTTGTTGG |  |
| **hsa-miR-125b-5p** | CCCTGAGACCCTAACTTGTG |  |
| **hsa-miR-29a-3p** | TAGCACCATCTGAAATCGGTTA |  |
